# Genome-wide identification and comparative analyses of key genes involved in C_4_ photosynthesis in five main gramineous crops

**DOI:** 10.3389/fpls.2023.1134170

**Published:** 2023-03-13

**Authors:** Liang Chen, Yang Yang, Zhangchen Zhao, Shan Lu, Qiumei Lu, Chunge Cui, Martin A. J. Parry, Yin-Gang Hu

**Affiliations:** ^1^ State Key Laboratory of Crop Stress Biology for Arid Areas, College of Agronomy, Northwest A&F University, Yangling, Shaanxi, China; ^2^ College of Agriculture, Shannxi Agricultural University (Institute of Crop Sciences), Taiyuan, Shanxi, China; ^3^ Lancaster Environment Centre, Lancaster University, Lancaster, United Kingdom; ^4^ Institute of Water Saving Agriculture in Arid Regions of China, Northwest A&F University, Yangling, Shaanxi, China

**Keywords:** Gramineae crops, C_4_ photosynthesis, C_3_ photosynthesis, Phylogenetic analysis, Expression pattern

## Abstract

Compared to C_3_ species, C_4_ plants showed higher photosynthetic capacity as well as water and nitrogen use efficiency due to the presence of the C_4_ photosynthetic pathway. Previous studies have shown that all genes required for the C_4_ photosynthetic pathway exist in the genomes of C_3_ species and are expressed. In this study, the genes encoding six key C_4_ photosynthetic pathway enzymes (β-CA, PEPC, ME, MDH, RbcS, and PPDK) in the genomes of five important gramineous crops (C_4_: maize, foxtail millet, and sorghum; C_3_: rice and wheat) were systematically identified and compared. Based on sequence characteristics and evolutionary relationships, their C_4_ functional gene copies were distinguished from non-photosynthetic functional gene copies. Furthermore, multiple sequence alignment revealed important sites affecting the activities of PEPC and RbcS between the C_3_ and C_4_ species. Comparisons of expression characteristics confirmed that the expression patterns of non-photosynthetic gene copies were relatively conserved among species, while C_4_ gene copies in C_4_ species acquired new tissue expression patterns during evolution. Additionally, multiple sequence features that may affect C_4_ gene expression and subcellular localization were found in the coding and promoter regions. Our work emphasized the diversity of the evolution of different genes in the C_4_ photosynthetic pathway and confirmed that the specific high expression in the leaf and appropriate intracellular distribution were the keys to the evolution of C_4_ photosynthesis. The results of this study will help determine the evolutionary mechanism of the C_4_ photosynthetic pathway in Gramineae and provide references for the transformation of C_4_ photosynthetic pathways in wheat, rice, and other major C_3_ cereal crops.

## 1 Introduction

Photosynthesis is one of the key biological innovations that allows organisms to convert light energy into chemical energy (ATP and NAD(P)H) for metabolic activities on a massive scale (Gest, 2002; Shih, 2015). During oxygenic photosynthesis, with the oxidation of water and the reduction of CO_2_, chemical energy generated from light energy is captured and used to synthesize organic compounds in higher plants in ‘dark reactions’ (Ashida et al., 2005; Paul, 2012; Blankenship, 2002). There are many different photosynthetic pathways in higher plants, the most widely known of which are the traditional C_3_, C_4_, and CAM (Crassulacean Acid Metabolism) types, and an in-depth study of the photosynthetic model species *Flaveria* identified the C_3_-C_4_ intermediate type (Moore et al., 1989).

Compared with C_3_ plants, C_4_ plants such as maize show higher photosynthetic capacity and greater water- and nitrogen-use efficiency, especially in suboptimal environments, which result in greater biomass production (Long, 1999; Paulus et al., 2013; Wang et al., 2014). Unlike C_3_ plants, which use the three-carbon molecule 3-phosphoglycerate (3-PGA) for carbon fixation, the carbon of C_4_ plants is first fixed in the four-carbon molecule oxaloacetate (OAA) in mesophyll cells (MCs) due to the presence of a CO_2_ concentrating mechanism (CCM). Immediately thereafter, OAA is transferred from the outer MCs to the bundle sheath cells (BSCs) in the form of malate (NADP-dependent malic enzyme subtype) or aspartate (NAD-dependent malic enzyme subtype). Then, CO_2_ is released in the chloroplasts of BSCs through decarboxylation by the malic enzyme, producing a microenvironment with a high CO_2_ concentration around the Rubisco enzyme (ribulose-1,5-bisphosphate carboxylase/oxygenase), thereby performing a Calvin cycle. The CCM enables Rubisco to function near its enzymatic V-max and greatly reduces the adverse effects of photorespiration, thus allowing for a greatly reduced investment of nitrogen in Rubisco proteins (Wang et al., 2014). These elegant biological innovations, including biochemical reactions (C_4_ carbon shuttle) and anatomical structure (Kranz anatomy), allow for more efficient water and nitrogen use by C_4_ plants in extreme climates (Hibberd and Covshoff, 2010).

C_4_ photosynthesis evolved from ancestral C_3_ photosynthesis during a global CO_2_ reduction and temperature increase (Sage et al., 2012). Most plants are C_3_ plants; however, it has been reported that the C_4_ pathway independently evolved in angiosperms, and the characteristics of this multisource evolution indicate that the transition of the photosynthetic pathway from the C_3_ pathway to the C_4_ pathway is relatively simple (Kellogg, 1999; Hibberd et al., 2008). In addition, some C_3_ plants exhibit C_4_ characteristics in specific environments, while C_4_ plants show C_3_ differentiation at specific growth stages, and some plants can convert between the C_3_ and C_4_ photosynthetic pathways, all of which indicate that the photosynthetic characteristics of C_3_ and C_4_ plants have great plasticity (Cheng et al., 1989; Ueno, 1998; Pyankov et al., 1999; Hibberd and Quick, 2002; Hibberd et al., 2008).

Gramineae is one of the most important model groups for world research because it contains a large number of important crop species. C_4_ grasses, including maize, sorghum, and foxtail millet, and C_3_ crops, such as wheat and rice, are all widely cultivated in modern agriculture and are major food crops critical to global food security (Leakey, 2009). The current annual low yield growth rates of wheat and rice have fallen far short of the target of doubling production by 2050 (Ray et al., 2013; FAO, 2020). Meanwhile, the increasing global population, unpredictable severe weather, and continuous reduction in water and arable land resources generate an extremely urgent need to advance main crop productivity (Parry et al., 2011). C_4_ photosynthetic transformation of C_3_ crops is the most likely method through which crop yield will be increased on a large scale to ensure global food security (von Caemmerer et al., 2012; Long et al., 2015). Engineering C_3_ food crops such as wheat and rice to use the C_4_ photosynthetic pathway has long been explored. To date, the identification and comparisons of C_4_ photosynthetic genes, their non-C_4_ types in C_4_ lineages and their close non-C_4_ relatives in Gramineae have been reported in several studies. Transcriptome comparison of 10 independent C_4_ origins and their 9 non-C_4_ relatives showed that the most highly expressed gene lineages in non-C_4_ ancestors may generate their C_4_ pathway by repeated co-optation (Moreno-Villena et al., 2017). Another comparative study, based on Gramineae C_4_ lineages and their non-C_4_ relatives, also confirmed that the same gene lineages were recruited in independent C_4_ origins despite the existence of multiple copies (Christin et al., 2013). Furthermore, previous studies based on single C_4_ photosynthetic key genes in several gramineous species, such as the carbonic anhydrase and phosphoenolpyruvate transporter genes, also confirmed that key genes are recruited into the C_4_ photosynthetic pathway by the acquisition of high expression levels and tissue expression characteristics (Ludwig, 2016; Lyu et al., 2020). All these results indicate that the recruitment and selection of C_4_ photosynthetic pathway genes are identical and affected by the expression abundance of gene lineages before C_4_ evolution. Therefore, accurate identification of C_4_ orthologues in C_3_ crops and comparative analysis with C_4_ genes in C_4_ crops are of great interest for understanding the evolution of the gramineous C_4_ pathway and improving the photosynthetic efficiency of C_3_ species.

In this study, the genes encoding the six key enzymes in the NADP-ME subtype of the C_4_ photosynthesis pathway in five important gramineous crops were identified and characterized. These genes were systematically classified into C_4_-type and non-photosynthetic gene copies based on their amino acid sequence characteristics and evolutionary relationships. In addition, their tissue expression patterns and sequence features in promoter regions were also compared, and a schematic diagram of the necessary steps for the transformation of the C_4_ photosynthetic pathway in gramineous C_3_ crops was proposed. The results will provide a foundation for understanding the C_4_ photosynthetic gene characteristics in Gramineae C_3_ and C_4_ crops.

## 2 Materials and methods

### 2.1 Identification of the genes encoding six C_4_ photosynthetic enzymes

Wheat genome and protein sequences (IWGSC v1.1) were obtained from the Wheat URGI database (Alaux et al., 2018; IWGSC, 2018), and the sequences of four other Gramineae crops (rice, foxtail millet, sorghum, and maize) were downloaded from the Ensemble Plants database (release 39, http://plants.ensembl.org). Then, six important enzymes (beta carbonic anhydrase, β-CA; ribulose bisphosphate carboxylase small subunit, RbcS; phosphoenolpyruvate carboxylase, PEPC; NADP-dependent malic enzyme, NADP-ME; malate dehydrogenase, MDH; and pyruvate, orthophosphate dikinase, PPDK) involved in the C_4_ photosynthetic pathway were identified (Kersey et al., 2015). First, five local protein databases were constructed using these sequences for homologous alignment of cloned sequences downloaded from the National Center for Biotechnology Information (NCBI, https://www.ncbi.nlm.nih.gov/) through a local protein basic local alignment search (BLASTP) program with an E-value cut-off < 10^-5^ and an identity of 60% as the threshold. The Hidden Markov Model (HMM) models of conserved domains of all six genes were obtained from the PFAM database (http://pfam.xfam.org/), and all genes predicted by BLASTP were further screened by their conserved domains using the HMMER search tool (Wheeler and Eddy, 2013). The NCBI-Conserved Domain Database (CDD) search was also used to check the conserved protein domains of these candidate genes (https://www.ncbi.nlm.nih.gov/cdd), and sequences lacking either of these domains were excluded. The PFAM ID and CDD Accession ID of the conserved domains of these genes are listed in **Table 1**. In addition, based on the understanding of the special dual promoter structure of *PPDK* genes, the gene structure annotations from NCBI (Maize, NCBI B73_v4 annotation release 102; Foxtail Millet, Setaria_italica_2.0 annotation release 103) were used to correct the *PPDK* genes from maize and foxtail millet. After manual curation, the nonredundant sequences were considered putative genes. Finally, gene duplication events of these genes in each species were determined by MCScanX, and manual screening was performed according to Wang et al. (2016).

**Table 1:** The number of genes encoding the six key enzymes involved in NADP-ME type C_4_ photosynthesis in five gramineous crops.

Target^a^	Number of copies					CDD_ID^b^	Pfam_ID^c^
	Rice	Foxtail Millet	Sorghum	Maize	Wheat		
β-CA	3	4	5	5	16	cl00391	PF00484
RbcS	4	5	1	2	24	cl01843	PF12338; PF00101
PEPC	5	5	5	4	15	cl21521	PF00311
NADP-ME	4	4	6	6	12	cl27704	PF03949; PF00390
MDH	10	11	10	13	26	cl27704	PF02866
PPDK	1	2	2	2	4	cl27021	PF02896; PF01326; PF00391
Total	27	31	29	32	97		

a β-CA, Beta carbonic anhydrase; RbcS, Ribulose bisphosphate carboxylase small subunit; PEPC, Phosphoenolpyruvate carboxylase; NADP-ME, NADP-dependent malic enzyme; MDH, Malate dehydrogenase; PPDK, Pyruvate, orthophosphate dikinase.

b The accession ID of conserved domains of these proteins in the NCBI – Conserved domain database (https://www.ncbi.nlm.nih.gov/cdd).

c The accession ID of conserved domains of these proteins in the PFAM database (http://pfam.xfam.org/).

### 2.2 Analysis of gene structure, conserved motifs, and the physical and chemical properties of encoded proteins

The gene structures were determined and displayed using the online tool Gene Structure Display Server (GSDS) 2.0 (http://gsds.cbi.pku.edu.cn/) (Guo et al., 2007). The multiple EM for motif elicitation (MEME) program (v5.5.0) was used to determine the conserved protein motifs of these genes, and the parameters were as follows: the optimal motif width was between 6 and 200 residues, allowing the presence of any number of repeating motif sites, and the maximum motif number was 20 (Bailey et al., 2009).

The biochemical parameters and subcellular localization were calculated by the Computer pI/MW tool in the ExPASy database (https://web.expasy.org/compute_pi/) (Gasteiger et al., 2005; Chou and Shen, 2008). The ChloroP server was used to predict the presence of chloroplast transit peptides (cTP) and the location of potential cTP cleavage sites (Emanuelsson et al., 1999). The protein sequences used to compare the differences between C_3_ and C_4_ plants were downloaded from the NCBI and UniProt databases (https://www.uniprot.org/).

### 2.3 Phylogenetic analysis and identification of C_4_-orthologous genes in C_3_ crops

To evaluate the evolutionary relationships among these genes, multiple sequence alignments were performed using the Clustal Omega program (https://www.ebi.ac.uk/Tools/msa/clustalo/) with the default parameters (Sievers et al., 2011). Phylogenetic analyses were conducted using both the neighbour-joining (NJ) method and maximum likelihood (ML) method. The ML trees were constructed using PhyML 3.1 (http://www.atgc-montpellier.fr/phyml/versions.php) with the JTT model. The NJ trees were constructed using MEGA6.06 with 1000 bootstrap replications, the JTT model, and the pairwise deletion option (Saitou and Nei, 1987; Tamura et al., 2013). The trees of these six genes were constructed using iTOL v6 software (https://itol.embl.de). In addition, MCscanX was used to compare the homology relationships between genes in different species (Wang et al., 2012).

### 2.4 Sequence analysis of the promoter region

The 2000 bp upstream sequence of the initiation codon was considered the promoter region for each gene and was extracted from the genome using the SAMtools program (v1.12) (Li, 2011). The cis-acting regulatory elements in the promoter region were predicted using PlantCARE (http://bioinformatics.psb.ugent.be/webtools/plantcare/html/) (Lescot et al., 2002). In addition, MEME was used to identify the conserved sequences in the promoter regions with an optimal motif width between 6 and 50 residues and a maximum motif number of 20.

### 2.5 Tissue-specific expression patterns

To investigate their expression profiles in different tissues, publicly available RNA-seq datasets of four tissues (root, shoot, spike, and leaf) in maize, sorghum, wheat, foxtail millet, and rice were downloaded from the following databases: the NCBI sequence read archive (SRA) (https://www.ncbi.nlm.nih.gov/sra), Gene Expression Omnibus (GEO) (https://www.ncbi.nlm.nih.gov/gds), Sorghum Functional Genomics Database (http://structuralbiology.cau.edu.cn/sorghum), *Setaria italica* Functional Genomics Database (http://structuralbiology.cau.edu.cn/SIFGD), MaizeGDB (https://www.maizegdb.org), expVIP (http://www.wheat-expression.com), and Rice Expression Database (http://expression.ic4r.org). In addition, the partial expression matrices of wheat, maize, and rice were directly obtained from public databases, and the raw RNA-seq data of sorghum (SRR959796, SRR959782, and SRR959765), foxtail millet (SRR442161-SRR442164) and rice (SRP028766 and SRP049212) were downloaded for reanalysis. The quality of raw reads was evaluated using FastQC v0.11.9, and the low-quality reads were filtered by Trimmomatic v0.36 (Andrews, 2010; Bolger et al., 2014). Clean reads were mapped onto their reference genomes using Hisat2 v2.2.1.0 (Pertea et al., 2016). The read numbers mapped to each gene were counted using HTSeq v0.11.1, and then the FPKM value of each gene was calculated based on the length of the gene and the read count mapped to the gene (Trapnell et al., 2010; Anders et al., 2015). To compare the tissue expression characteristics of homologous genes in different species, EL (expression level) values were used to draw a heatmap to display the tissue-specific patterns of expression. EL values were calculated using the following formula:


$$\text{Expression Level }\left(\text{EL}\right)=\frac{-\text{log}2\left(\text{FPKM}+1\right)}{-\text{log}2\left(\text{sum}\left(\text{FPKM}+1\right)\right)}\times 100\%$$

where the EL value represents the expression in a certain tissue in proportion to that in all tissues and FPKM represents fragments per kilobase of exon model per million mapped fragments.

## 3 Results

### 3.1 Identification of the genes encoding six key C_4_ photosynthetic enzymes

The six key C_4_ photosynthetic enzymes (β-CA, RbcS, PEPC, NADP-ME, MDH, and PPDK) are all encoded by small family genes. The availability of the genome sequences of the five gramineous crops made it possible to identify all family members of the six key enzymes, including C_4_ and non-photosynthetic gene copies. A total of 216 genes with complete conserved domains were identified in five crop genomes (**Table 1**, **Tables S2–S7**). The MDH family was the largest, and the PPDK family had the fewest members among the six gene families of the five Gramineae crop genomes. For the convenience of description, all genes were renamed based on their homologous relationships. In short, the orthologous genes with high homology among the five crops were first numbered, and then the paralogous genes in each species were numbered. For example, β-CA3 enzymes were encoded by seven genes, namely, *SiCA3*, *ZmCA3*, *SbCA3*, *TaCA3-3A*, *TaCA3-3B*, *TaCA3-3D* and *OsCA3*, while β-CA5 enzymes were encoded by only three genes in wheat, *TaCA5-7A*, *TaCA5-7B* and *TaCA5-7D* (**Table 2**). Compared with other crops, wheat possessed the most copies as a result of its allohexaploid genome and complex evolutionary process.

**Table 2:** *β-CA*, *PEPC*, *ME*, and *MDH* genes involved in the C_4_ photosynthesis pathway in five gramineous crops.

Target^a^	Name	Foxtail Millet	Sorghum	Maize	Rice	Wheat		
						A-subgenome	B-subgenome	D-subgenome
*β-CA*	*β-CA1*	*KQL24850*	*KXG35798*	*Zm00001d020764*, *Zm00001d005920*	*Os09t0464000*	*TraesCS5A02G245700*	*TraesCS5B02G243100*	*TraesCS5D02G252400*
	*β-CA2*	*KQL06053*	*KXG32978*	*—*	*Os01t0640000*	*TraesCS3A02G230100*	*TraesCS3B02G259400*	*TraesCS3D02G223200*
	***β-CA3***	***KQL06049***	***KXG32970***	***Zm00001d044099***	***Os01t0639900***	***TraesCS3A02G230000***	***TraesCS3B02G259300***	***TraesCS3D02G223300***
	*β-CA4*	*KQL06051*	*KXG32975*	*Zm00001d044095*	*—*	*—*	*—*	*—*
	*β-CA5*	*—*	*—*	*—*	*—*	*TraesCS7A02G454300*	*TraesCS7B02G354800*	*TraesCS7D02G443400*
	*β-CA6*	*—*	*—*	*—*	*—*	*TraesCS7A02G454500*	*TraesCS7B02G354900*	*TraesCS7D02G443500*
	*β-CA7*	*—*	*—*	*—*	*—*	*TraesCS7A02G454400*	*—*	*—*
	*β-CA8*	*—*	*KXG32973*	*—*	*—*	*—*	*—*	*—*
	*β-CA9*	*—*	*—*	*Zm00001d044096*	*—*	*—*	*—*	*—*
	*β-CA10*	*—*	*—*	*Zm00001d011454*	*—*	*—*	*—*	*—*
*PEPC*	*PEPC1*	*SETIT_028826mg*	*SORBI_3002G167000*	*Zm00001d020057*	*Os09t0315700*	*TraesCS5A02G181800*	*TraesCS5B02G179800*	*TraesCS5D02G186200*
	*PEPC2*	*SETIT_000184mg*	*SORBI_3003G301800*	*Zm00001d012702*	*Os01t0758300*	*TraesCS3A02G306700*	*TraesCS3B02G329800*	*TraesCS3D02G295200*
	*PEPC3*	*SETIT_016228mg*	*SORBI_3004G106900*	*Zm00001d053453*	*Os02t0244700*	*TraesCS6A02G195600*	*TraesCS6B02G223100*	*TraesCS6D02G183200*
	***PEPC4***	***SETIT_005789mg***	***SORBI_3010G160700***	***Zm00001d046170***	***—***	***TraesCS7A02G345400***	***TraesCS7B02G237900***	***TraesCS7D02G333900***
	*PEPC5*	*SETIT_000160mg*	*—*	*—*	*Os01t0208700*	*TraesCS3A02G134200*	*TraesCS3B02G168000*	*TraesCS3D02G150500*
	*PEPC6*	*—*	*SORBI_3007G106500*	*—*	*Os08t0366000*	*—*	*—*	*—*
*ME*	*ME1*	*SETIT_000808mg*	*SORBI_3003G292400*	*Zm00001d012764*	*Os01t0743500*	*TraesCS3A02G275600*	*TraesCS3B02G309300*	*TraesCS3D02G275500*
	*ME2*	*SETIT_000774mg*	*SORBI_3003G280900*	*Zm00001d043601*	*Os01t0723400*	*TraesCS3A02G285900*	*TraesCS3B02G320200*	*TraesCS3D02G285700*
	*ME3*	*SETIT_021600mg*	*SORBI_3009G069600*	*Zm00001d037693*	*Os05t0186300*	*TraesCS1A02G122500*	*TraesCS1B02G141700*	*TraesCS1D02G123400*
	***ME4***	***SETIT_000645mg***	***SORBI_3003G036200***	***Zm00001eb121470***	***Os01t0188400***	***TraesCS3A02G108900***	***TraesCS3B02G128000***	***TraesCS3D02G110700***
	*ME5*	*—*	*SORBI_3003G036000*	*Zm00001d037961*	*—*	*—*	*—*	*—*
	*ME6*	*—*	*—*	*Zm00001d037962*	*—*	*—*	*—*	*—*
	*ME7*	*—*	*—*	*Zm00001d010358*	*—*	*—*	*—*	*—*
*MDH*	*MDH1*	*SETIT_036550mg*	*SORBI_3001G219300*	*Zm00001d032695*	*Os10t0478200*	*TraesCS1A02G155200*	*TraesCS1B02G172400*	*TraesCS1D02G153900*
	*MDH2*	*SETIT_010442mg*	*SORBI_3006G170800*	*Zm00001d002741*	*Os04t0551200*	*—*	*—*	*—*
	***MDH3***	***SETIT_013632mg***	***SORBI_3007G166200***	***Zm00001d031899***	***Os08t0562100***	***TraesCSU02G135100***	***TraesCS7B02G197000***	***TraesCS7D02G283900***
	*MDH4*	*SETIT_013547mg*	*SORBI_3007G137600*	*Zm00001d032187*	*Os08t0434300*	*TraesCS1A02G348500*	*TraesCS1B02G363100*	*TraesCS1D02G351500*
	*MDH5*	*SETIT_030117mg*	*OQU90313*	*Zm00001d022229*	*Os07t0630800*	*—*	*—*	*—*
	*MDH6*	*SETIT_022438mg*	*SORBI_3008G186200*	*Zm00001d041243*	*Os12t0632700*	*TraesCS5A02G014300*	*TraesCS5B02G012400*	*TraesCS5D02G019700*
	*MDH7*	*SETIT_036365mg*	*SORBI_3001G073900*	*Zm00001d034241*	*Os03t0773800*	*TraesCS5A02G407700*	*TraesCS5B02G412500*	*TraesCS5D02G417600*
	*MDH8*	*SETIT_022574mg*	*SORBI_3009G240700*	*Zm00001d039089*	*Os05t0574400*	*TraesCS1A02G412900*	*TraesCS1B02G443200*	*TraesCS1D02G420500*
	*MDH9*	*SETIT_002110mg*	*SORBI_3003G238500*	*Zm00001d044042*	*Os01t0649100*	*TraesCS3A02G234800*	*TraesCS3B02G265000*	*TraesCS3D02G236200*
	*MDH10*	*—*	*—*	*Zm00001d014030*	*—*	*—*	*—*	*—*
	*MDH11*	*—*	*—*	*Zm00001d019330*	*—*	*—*	*—*	*—*
	*MDH12*	*—*	*—*	*—*	*—*	*TraesCS5A02G549900*	*TraesCSU02G127700*	*—*
	*MDH13*	*SETIT_029817mg*	*—*	*—*	*—*	*—*	*—*	*—*
	*MDH14*	*—*	*SORBI_3007G166300*	*—*	*—*	*—*	*—*	*—*
	*MDH15*	*—*	*—*	*—*	*—*	*TraesCS7A02G386600*	*TraesCS7B02G289400*	*TraesCS7D02G383100*
	*MDH16*	*—*	*—*	*—*	*Os01t0829800*	*—*	*—*	*—*
	*MDH17*	*SETIT_022252mg*	*—*	*—*	*—*	*—*	*—*	*—*
	*MDH18*	*—*	*—*	*Zm00001d050409*	*—*	*—*	*—*	*—*
	*MDH19*	*—*	*—*	*Zm00001d009640*	*—*	*—*	*—*	*—*

a β-CA4, PEPC4, ME4 and MDH3 were C_4_-type copies (Bold).

Although the number of these genes encoding key C_4_ photosynthetic enzymes in different species was not identical, except for *RbcS*, the number and distribution of the other five gene family members showed similar trends in the C_3_ and C_4_ crop genomes (**Table 1**). The copy number of the *RbcS* gene in different grass species showed a large difference, and there were 24 *RbcS* copies in wheat and only one copy in sorghum. In addition, 30 of the 36 *RbcS* genes were associated with tandem duplication events, accounting for 83.33% of all *RbcS* genes (**Table S3**). Similarly, tandem duplicated genes accounted for 73.53% (25/34) of all *β-CA* genes (**Table 2**, **Table S2**).

Earlier reports indicate that the subcellular-specific expression of the *PPDK* gene is regulated by a dual promoter located upstream of the first exon and on the first intron (Sage et al., 2012). The first exon encodes a chloroplast transit peptide; the copy regulated by the first promoter exhibits a chloroplast type, and the copy regulated by the second promoter exhibits a cytoplasmic type. However, our results showed that maize has two chloroplast copies, while foxtail millet has two mitochondrial copies. To further explain this discrepancy, the NCBI-annotated maize genome sequence (Maize, NCBI B73_v4 annotation release 102, ftp://ftp.ncbi.nlm.nih.gov/genomes/Zea_mays) and foxtail millet genome sequence (Foxtail Millet, NCBI Setaria_italica_v2.0 annotation release 103, ftp://ftp.ncbi.nlm.nih.gov/genomes/Setaria_italica) were downloaded and analysed. Further alignment analysis revealed that *Zm00001d010321* (*ZmPPDK2*) in the current genome (Zm-B73-REFERENCE-GRAMENE-4.0) had a false annotation, and this gene (*LOC103635678; NM_001358399.1*) annotated by NCBI was shorter in length and lacked the first exon sequence, proving that *ZmPPDK2* encoded a cytoplasmic PPDK isoform (**Table S7**). Similarly, *SiPPDK1.1* (*LOC101760933*; *XM_004962073.4*) encoded a 945 aa chloroplast PPDK, which was significantly different from SETIT_021174mg, encoding an 889 a.a. cytoplasmic PPDK (**Table S7**). In general, all genes encoding six key C_4_ photosynthetic enzymes were identified completely and accurately in these five Gramineae species.

### 3.2 Phylogenetic analysis and identification of C_4_-orthologous genes in C_3_ crops

**Figure 1:**
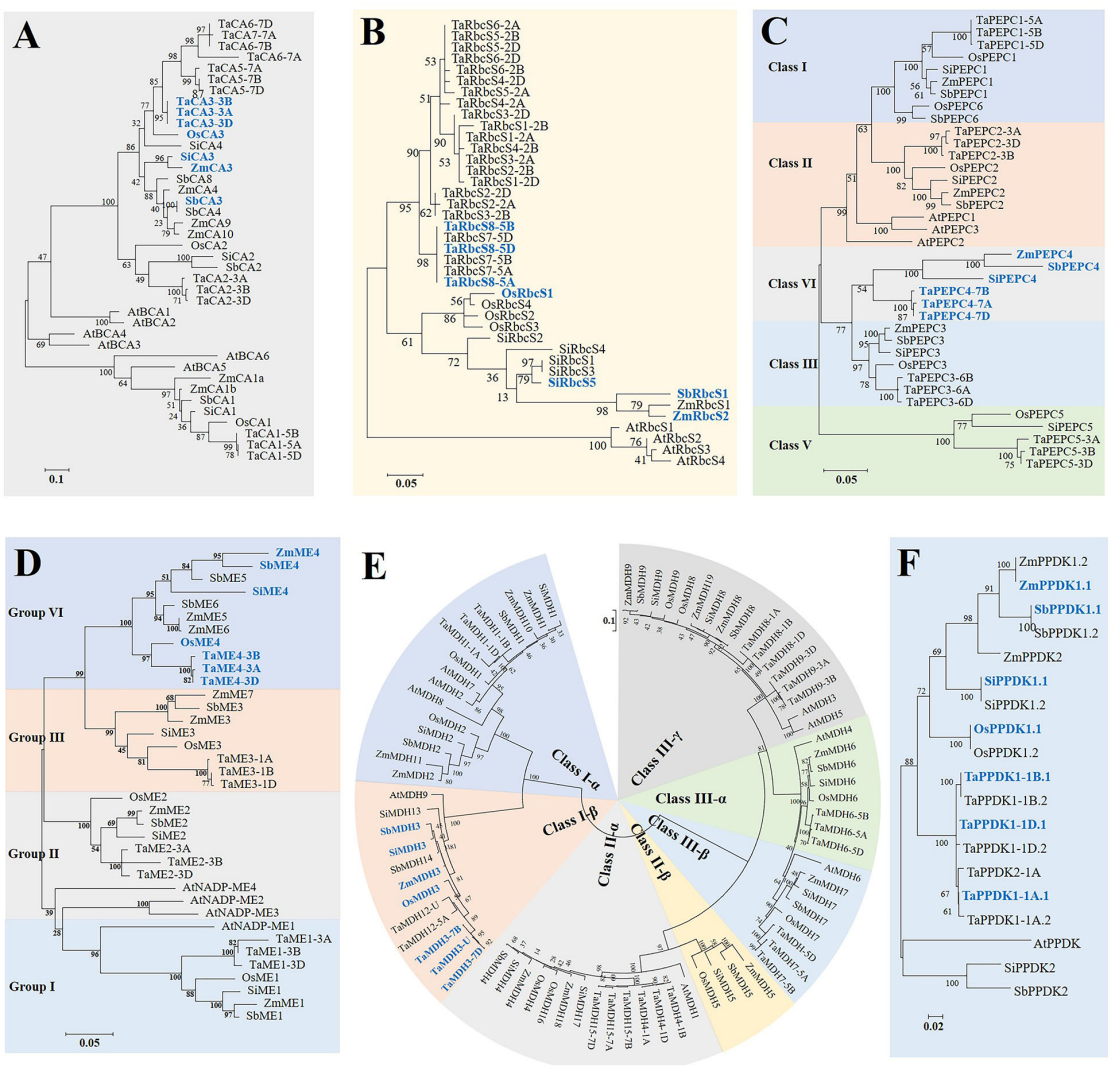
Phylogenetic relationships between six gene families involved in the C_4_ photosynthetic pathway from common wheat (Ta), maize (Zm), foxtail millet (Si), rice (Os), sorghum (Sb), and *Arabidopsis* (At) based on their amino acid sequences. Multiple sequence alignment and phylogenetic tree construction were performed using MUSCLE and MEGA 6.0 (maximum likelihood method, JTT model), respectively. Scale bars indicate the number of amino acid substitutions per site. Genes presented in bold blue fonts represent C_4_-type copies in C_4_ species. **(A)** β-CA; **(B)** RbcS; **(C)** PEPC; **(D)** NADP-ME; **(E)** MDH; **(F)** PPDK.

The full-length amino acid sequences of these identified genes were aligned, and then phylogenetic trees were constructed using both the maximum likelihood (ML) and neighbour-joining (NJ) methods. NJ trees and ML trees of different genes all showed very consistent topological structures (**Figure 1**, **Figure S1**). Except for RbcS and PPDK, the other genes could be divided into three to five distinct lineage homologous branches, including three for β-CA, five for PEPC, four for ME, and four for MDH, as detailed below.

#### 3.2.1 Beta carbonic anhydrase (β-CA)

**Figure 2:**
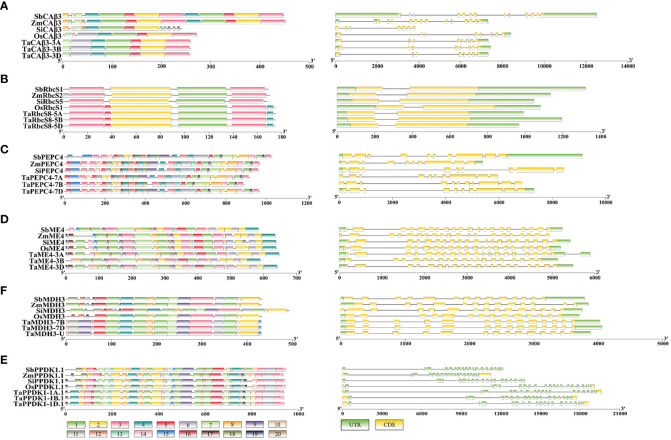
The gene structures and conserved protein domains of putative C_4_/C_4_-homologous genes in five gramineous crops. **(A–E)** represent β-CA, RbcS, PEPC, ME, MDH, and PPDK, respectively. The graph on the left is the distribution of conserved motifs on these proteins, and the right panel is the gene structure.

The β-CA genes in the five crops were clustered into three major groups, of which β-CA1 (8), β-CA2 (6), and other members (20) clustered together into one category. The β-CA1 and β-CA2 branches were relatively conserved and evenly distributed in each species, while the third branch produced more copy number variations due to tandem duplication events (**Figure 1A**, **Table S2**). All *β-CA3*, *β-CA4*, and *β-CA5* genes from the same species were tandem duplicated genes, such as *TaCA5-7A*, *TaCA6-7A*, and *TaCA7-7A*. This indicated that tandem duplication was an important mechanism for the expansion and functional diversification of the β-CA family, and the third branch was an important source of new functional generation. In addition, except for TaCA7, which only existed in the wheat A subgenome, other TaCAs had paralogous copies in the A, B, and D subgenomes, confirming that TaCAs were relatively conserved during wheat polyploidization (**Figure 2A**).

**Figure 3:**
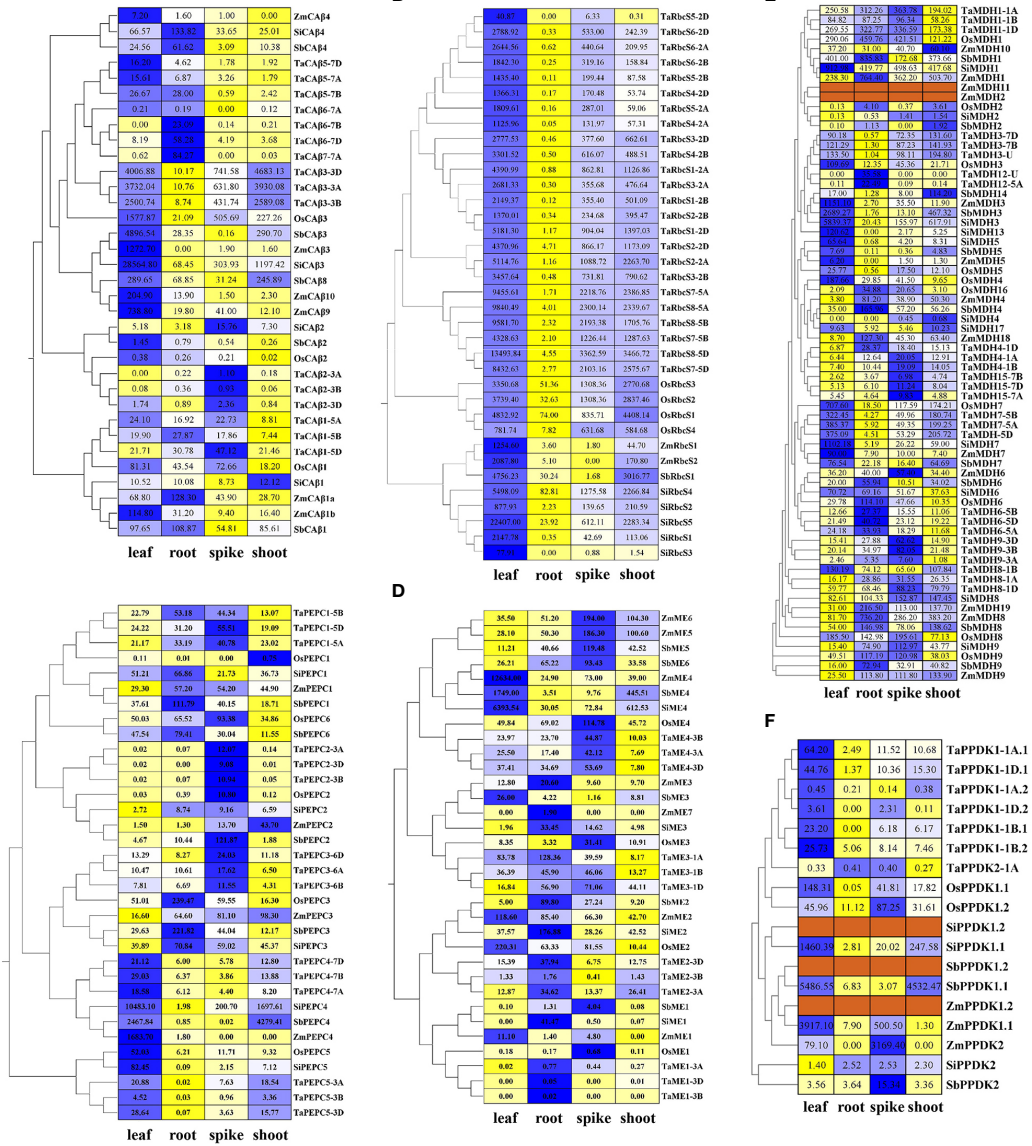
Expression levels of all putative genes encoding key C_4_ photosynthetic enzymes in four tissues (leaf, root, stem, and spike) from RNA-seq data. The heatmaps were drawn based on the EL values after the normalization of different tissues, with their original FPKM values listed in the corresponding grid. All genes were clustered according to their phylogenetic relationship. The blue and yellow colours represent higher and lower relative abundance for each transcript in each sample, respectively, and the dark red colour represents no data support. Based on the special double promoter structure of PPDK, the expression levels of all transcripts are listed. **(A)** *β-CA*; **(B)** *RbcS*; **(C)** *PEPC*; **(D)** *ME*; **(E)** *MDH*; **(F)** *PPDK*.

Phylogenetic analysis of the β-CA genes showed that TaCA3, OsCA3, SiCA3, SbCA3, and ZmCA3 were clustered together, and collinearity analysis based on MCscanX also showed that these genes were orthologous (**Figure 1A**, **Table S2**). In addition, the β-CA3 genes of the C_4_ species were highly expressed in leaves; therefore, they were determined to be gene copies involved in C_4_ photosynthesis (**Figure 3A**).

#### 3.2.2 Ribulose bisphosphate carboxylase small subunit (RbcS)

The phylogenetic tree showed that *RbcS* genes of the same species were clustered into one class, which confirmed that the expansion of the RbcS family occurred after the division of the grass species (**Figure 1B**). Since the expansion of the RbcS family occurred after species differentiation, it is relatively difficult to identify its orthologous genes. Therefore, we believed that gene copies with specifically high expression in leaves were predominantly *RbcS* genes. Finally, based on their leaf-specific high expression, *TaRbcS8*, *OsRbcS1*, *SbRbcS1*, *ZmRbcS2*, and *SiRbcS5* were considered putative genes encoding the ribulose bisphosphate carboxylase small subunit (**Figure 3B**).

#### 3.2.3 Phosphoenolpyruvate carboxylase (PEPC)

Phylogenetic analysis showed that PEPC genes were divided into five classes, which contained 9, 7, 7, 6, and 5 members (**Figure 1C**). The first three groups each contained at least one PEPC gene copy from each species (PEPC1, PEPC2, and PEPC3), which indicated that the function of PEPC in these three groups should be relatively conserved. The fifth class contained only PEPC gene copies from wheat, rice, and foxtail millet. PEPC genes in the fifth class should not perform the C_4_ function. In addition, the PEPC genes of each group in wheat contained three copies from the A, B, and D subgenomes.

Previous studies on C_3_ and C_4_ *Flaveria* species indicated that the serine (S, Ser) residue in the C-terminus is an important symbol of C_4_ PEPC isoforms (Bläsing et al., 2002; Gowik and Westhoff, 2011). The same serine residue was found in three PEPC4 copies from C_4_ crops (at position 780 of ZmPEPC4), which suggested that the PEPC4 isoforms were C_4_-type, and this was confirmed by their high expression in leaves (**Figure 3C**).

#### 3.2.4 NADP-dependent malic enzyme (NADP-ME)

ME genes of five crops were clustered into four groups, each containing 7, 7, 8, and 11 genes. Similar to PEPC genes, ME genes from C_4_ and C_3_ crops were evenly distributed in the first three groups (ME1, ME2, and ME3), while more genes from C_4_ species were included in the fourth group (ZmME4-6, SbME4-6, SiME4, OsME4, and TaME4), which suggested that there might be more functional differentiation in the fourth group (**Figure 1D**).

Previous studies have shown that the evolution of C_4_ *NADP-ME* originates from the acquisition of the chloroplast transit peptide of the cytosolic ancestor and subsequent new functionalization, and C_4_ *NADP-ME* is specifically located in the chloroplast (Tausta et al., 2002). Only *ME* gene copies in the fourth group were found to be chloroplast type, and transcriptome data also confirmed that *ME4* genes from C_4_ species were highly expressed in leaves, indicating that *ME4s* function in the plant C_4_ photosynthetic pathway (**Figure 3D**, **Table S5**).

#### 3.2.5 Malate dehydrogenase (MDH)

Multiple forms of MDHs are present in higher plants with different subcellular localizations and coenzyme specificities. Based on the difference in coenzymes, they can be divided into two types, NAD-dependent MDHs (NAD-MDHs) and NADP-dependent MDHs (NADP-MDHs). NADP-MDHs are mainly present in chloroplasts and cytoplasm, while NAD-MDHs are distributed in mitochondria, the cytoplasm, peroxisomes, and plastids (Gietl, 1992). In C_4_ photosynthesis, the chloroplastic NADP-MDH present in MCs converts OAA into malic acid to perform the C_4_ function. Based on the prediction of subcellular localization and the coenzyme-specific binding site (at position 43 of OsMDH1, D and G for NAD-dependent MDH and NADP-dependent MDH, respectively), these MDHs were well classified (**Table S6**).

The phylogenetic tree showed that all different types of MDHs could be assigned to different branches (**Figure 1E**, **Table S6**). MDH1, MDH2, ZmMDH10, and ZmMDH11 were NAD-dependent cytoplasmic isoforms, and MDH6 and MDH7 were NAD-dependent peroxisome isoforms. In terms of evolutionary relationships, cytoplasmic NAD-MDHs and chloroplast NADP-MDHs (MDH3, SiMDH13, SbMDH14, and TaMDH12) were located in one branch, which indicated that chloroplast NADP-MDHs are more closely related to cytoplasmic NAD-MDHs. The phylogenetic data combined with the finding of ultrahigh expression levels of *MDH3* genes in the leaves of three C_4_ crops, indicated that *MDH3* genes are C_4_ photosynthetic gene copies (**Figure 3E**). Although both *SiMDH13* and *SbMDH14* were located in the same branch as *MDH3* genes, neither of them exhibited leaf-specific high expression and were therefore excluded.

#### 3.2.6 Pyruvate, orthophosphate dikinase (PPDK)

The *PPDK* gene directs the transcription of different subcellular-targeted PPDK isoforms through a specific dual-promoter structure, so different subcellular-localized PPDK proteins (PPDK1.1 and PPDK1.2) encoded by *PPDK1* were also used to construct the phylogenetic tree (**Figure 1F**). Eleven genes encoding PPDK were identified in five species, of which two genes were identified in all C_4_ species, while only one gene was identified in rice, and four genes were identified in wheat. Using multiple sources of genome information to study *PPDK* genes in maize and foxtail millet, it was confirmed that PPDK1 contained a dual-promoter structure and was transcribed to a chloroplast PPDK isoform and a cytoplasmic PPDK isoform, while *PPDK2* encoded another cytoplasmic PPDK isoform. *PPDK1* was highly expressed in the leaves of all C_4_ crops, while rice and wheat lacked some *PPDK2* copies, so PPDK1 was identified as a C_4_ type gene (**Figure 3F**, **Table S7**).

### 3.3 Comparative analysis of sequence variations among C_4_ and C_4_ orthologue gene copies in C_4_ and C_3_ crops

Based on the prediction of protein conserved domains by MEME software, the annotation information of gene structure, the sequence, and their physicochemical characteristics, differences between C_4_ genes in C_4_ species and C_4_-orthologous genes in C_3_ crops were compared in detail.

#### 3.3.1 Carbonic anhydrase β type (β-CA)

The gene structures of *β-CA3* of C_3_ and C_4_ crops showed large differences, but they were relatively conserved among crops with the same photosynthetic type (**Figure 2A**). *TaCA3* and *OsCA3* genes both contained 8 exons, while *SiCA3*, *ZmCA3*, and *SbCA3* contained 7, 13, and 13 exons, respectively. In general, for all *CA3 genes*, the sequences from exon 1 to exon 7 were similar, although the lengths of these exons were different in C_3_ and C_4_ crops. For the conserved domains, a significant difference was that C_4_ β-CA3s lacked motif 6, while C_4_- orthologous β-CA3s contained motif 8 at their N-terminus in C_3_ crops (**Figure 2A**). Prediction of chloroplast transit peptides (cTPs) revealed that all TaCA3s had cTPs of approximately 48 amino acid residues at the N-terminus (TaCA3-3A, 49; TaCA3-3B, 48; TaCA3-3D, 48), and OsCA3 had a cTP of 68 amino acid residues, whereas all β-CA3s of C_4_ crops did not have cTPs (**Figure S2**). This was consistent with the position of motif 7-motif 6, confirming that C_4_ β-CA3s could fix CO_2_ in the cytoplasm by the deletion of motif 6 (Ludwig, 2016). In addition, the deletion of motif 6 improved the isoelectric point (pI) of C_4_ β-CA3s, which ranged from 8.74 to 8.80, while the pI of C_4_-homologous β-CA3s ranged from 8.35 to 8.41 (**Table 3**).

**Table 3:** Characteristics of the putative C_4_/C_4_-homologous genes involved in C_4_ photosynthesis in five gramineous crops.

Genes	Species	Gene ID	Gene ID	Protein ID	Location	AA Length	Molecular Weight	Subcellular Location^a^	pI	High Expression Tissue
β-CA	Sorghum	SbCA3	SORBI_3003G234200	KXG32970	Chr3:57297987-57310502	448aa	48986.93	cytoplasm	8.74	leaf shoot
	Maize	ZmCA3	Zm00001d044099	Zm00001d044099_P002	Chr3:219075378-219080211	452aa	49363.10	cytoplasm	8.74	leaf
	Millet	SiCA3	SETIT_003882mg	KQL06049	Chr5:30333130-30336956	336aa	26378.56	cytoplasm	8.80	leaf shoot spike
	Rice	OsCA3	Os01g0639900	Os01t0639900-01	Chr1:25696671-25705077	272aa	29117.42	chloroplast	8.41	leaf spike shoot
	Wheat	TaCA3-3A	TraesCS3A02G230000	TraesCS3A02G230000.2	Chr3A:430330494-430337814	259aa	28076.34	chloroplast	8.35	leaf shoot spike
	Wheat	TaCA3-3B	TraesCS3B02G259300	TraesCS3B02G259300.1	Chr3B:417258446-417265886	258aa	27963.18	chloroplast	8.35	leaf shoot spike
	Wheat	TaCA3-3D	TraesCS3D02G223300	TraesCS3D02G223300.1	Chr3D:304327703-304335024	258aa	27977.21	chloroplast	8.35	leaf shoot spike
RbcS	Sorghum	SbRbcS1	SORBI_3005G042000	EES09292	Chr5:3876057-3877378	169aa	19058.87	Chloroplast	8.77	leaf
	Maize	ZmRbcS2	Zm00001d052595	Zm00001d052595_T001	Chr4:194257728-194258862	170aa	19150.99	Chloroplast	9.10	leaf
	Millet	SiRbcS5	SETIT_023465mg	KQL15806	Chr3:24102022-24103068	169aa	19082.08	Chloroplast	8.64	leaf
	Rice	OsRbcS1	Os12g0274700	Os12t0274700-01	Chr12:10080505-10081588	175aa	19646.68	Chloroplast	9.04	leaf
	Wheat	TaRbcS8-5A	TraesCS5A02G165700	TraesCS5A02G165700.1	Chr5AL:354314588-354315579	175aa	19490.51	Chloroplast	8.80	leaf
	Wheat	TaRbcS8-5B	TraesCS5B02G162800	TraesCS5B02G162800.1	Chr5BL:300097036-300098231	175aa	19490.51	Chloroplast	8.80	leaf
	Wheat	TaRbcS8-5D	TraesCS5D02G169900	TraesCS5D02G169900.2	Chr5DL:266325112-266326079	175aa	19490.51	Chloroplast	8.80	leaf
PEPC	Sorghum	SbPEPC4	SORBI_3010G160700	EER89889	Chr10:47244445-47253575	1028aa	115731.7	Chloroplast	6.12	leaf
	Maize	ZmPEPC4	Zm00001d046170	Zm00001d046170_P001	Chr9:68851094-68856482	970aa	109341	Chloroplast	5.73	leaf
	Millet	SiPEPC4	SETIT_005789mg	KQL10919	Chr4:28034710-28043142	964aa	109982.7	Chloroplast	5.95	leaf
	Wheat	TaPEPC4-7A	TraesCS7A02G345400	TraesCS7A02G345400.1	Chr7AL:507757190-507763146	919aa	104542.4	Chloroplast	5.90	leaf
	Wheat	TaPEPC4-7B	TraesCS7B02G237900	TraesCS7B02G237900.1	Chr7BL:443292417-443299286	892aa	101122.3	Chloroplast	5.65	leaf
	Wheat	TaPEPC4-7D	TraesCS7D02G333900	TraesCS7D02G333900.1	Chr7DL:425502517-425509832	968aa	109614.9	Chloroplast	5.66	leaf
ME	Sorghum	SbME4	SORBI_3003G036200	EES00150	Chr3:3317184-3322427	636aa	69377.63	chloroplast	5.49	leaf
	Maize	ZmME4	GRMZM2G085019	GRMZM2G085019_P01	Chr3:7276387-7281737	636aa	69818.85	Chloroplast	6.20	leaf
	Millet	SiME4	SETIT_000645mg	KQL04777	Chr5:11687298-11692722	639aa	70037.89	chloroplast	6.31	leaf
	Rice	OsME4	Os01g0188400	Os01t0188400-01	Chr1:4739271-4744472	639aa	69865.84	chloroplast	6.70	spike, root
	Wheat	TaME4-3A	TraesCS3A02G108900	TraesCS3A02G108900.1	Chr3AL:74781464-74787353	648aa	70942.07	chloroplast	6.64	spike, leaf
	Wheat	TaME4-3B	TraesCS3B02G128000	TraesCS3B02G128000.1	Chr3BS:106962508-106968100	591aa	65203.5	chloroplast	5.86	spike, leaf
	Wheat	TaME4-3D	TraesCS3D02G110700	TraesCS3D02G110700.1	Chr3DL:64370009-64375502	642aa	70286.32	chloroplast	6.64	spike, leaf
MDH	Sorghum	SbMDH3	SORBI_3007G166200	KXG25371	Chr7:60144110-60147894	434aa	46880.56	chloroplast	5.51	leaf
	Maize	ZmMDH3	Zm00001d031899	Zm00001d031899_P002	Chr1:205992187-205996030	432aa	46786.63	chloroplast	6.49	leaf
	Millet	SiMDH3	SETIT_013632mg	KQL03004	Chr6:35757135-35760887	493aa	52791.61	chloroplast	6.24	leaf
	Rice	OsMDH3	Os08g0562100	Os08t0562100-01	Chr8:28141176-28144882	433aa	47008.9	chloroplast	6.96	leaf
	Wheat	TaMDH3-7B	TraesCS7B02G197000	TraesCS7B02G197000.2	Chr7BL:339613762-339617788	431aa	46682.5	chloroplast	6.62	leaf
	Wheat	TaMDH3-7D	TraesCS7B02G197000	TraesCS7D02G283900.1	Chr7DS:296507630-296511690	431aa	46814.67	chloroplast	6.96	leaf
	Wheat	TaMDH3-U	TraesCSU02G135100	TraesCSU02G135100.2	ChrUn:116430718-116434600	431aa	46814.67	chloroplast	7.52	leaf
PPDK	Sorghum	SbPPDK1.1	SORBI_3009G132900	KXG21962	Chr9:48726358-48738528	948aa	102490.21	chloroplast	5.54	leaf
	Maize	ZmPPDK1.1	Zm00001d038163	Zm00001d038163_P002	Chr6:150024486-150035717	936aa	101251	chloroplast	5.76	leaf
	Millet	SiPPDK1.1	LOC101760933	XP_004962130.1	Chr3:21258962-21273297	945aa	102424.94	chloroplast	5.38	leaf
	Rice	OsPPDK1.1	Os05g0405000	Os05t0405000-01	Chr5:19718538-19737605	947aa	102787.88	chloroplast	5.98	leaf
	Wheat	TaPPDK1-1A.1	TraesCS1A02G253400	TraesCS1A02G253400.1	Chr1AL:445257710-445277310	939aa	101861.56	chloroplast	5.64	leaf
	Wheat	TaPPDK1-1B.1	TraesCS1B02G264900	TraesCS1B02G264900.1	Chr1BL:465683651-465701383	939aa	101950.77	chloroplast	5.68	leaf
	Wheat	TaPPDK1-1D.1	TraesCS1D02G252900	TraesCS1D02G252900.1	Chr1DL:345264118-345282757	939aa	101845.56	chloroplast	5.64	leaf

a : The subcellular localization of proteins was predicted using LocTree3 (https://rostlab.org/services/loctree3/).

#### 3.3.2 Ribulose bisphosphate carboxylase small subunit (RbcS)

C_4_ and C_4_-homologous *RbcS* genes did not differ significantly in gene structure, and both contained two exons (**Figure 2B**). In contrast, two additional motifs were observed in C_4_-homologous RbcSs in C_3_ species, motif 5 and motif 4, located near the N-terminus and at the C-terminus, respectively. Comparison of the mutation sites between C_4_-homologous and C_4_ RbcS proteins found variations at multiple sites, such as (T/S)24G and N56I on OsRbcS1 (**Figure S3**). To identify the specificity of these sites, more RbcS protein sequences of Gramineae species were downloaded and compared, including 172 sequences from 40 species of 18 genera. The first site (24 on OsRbcS1) was found to be highly conserved with a G (glycine) or D (aspartate) among C_3_ species, while it showed differences among C_4_ species, T (threonine) for *Sorghum bicolor* and maize, S (serine) for *Saccharum hybrid*, G (glycine) and R (arginine) for foxtail millet, and G (glycine) for *Echinochloa crusgalli* and *Panicum hallii* (**Table 4**). Considering that the two species only have one protein sequence and considering the close relationship between grass and rice, *Panicum hallii*, and *Echinochloa crusgalli*, it is believed that there is a non-G site RbcS isoform in these two species. The second site (56 on OsRbcS1) could strictly distinguish the C_4_ and C_3_ species in all Gramineae RbcS sequences obtained, with N (asparagine) for C_4_ species and I (isoleucine) for C_3_ species. In addition, the prediction of protein structure suggested that the 8 amino acid residues at positions 51-59 of OsRbcS1 constituted a protein binding site, and the flanking sequences were highly conserved, so the variation at position 56 may result in important differences in function.

**Table 4:** Amino acids (10^th^ position, bold) for C_4_-specific RbcS across 40 species from 18 genera of Gramineae.

Genus	Species	Amino acid with Flanking region^a^	Amino acid with Flanking region^b^	P-type^c^
*Echinochloa*	*Echinochloa crus-galli*	PFQGLKSTA**G**L	CMQIWPVEN**N**	C4
*Saccharum*	*Saccharum hybrid*	PFQGLKSTA**S**L	CMQVWPAYG**N**	C4
*Sorghum*	*Sorghum bicolor*	PFQGLKSTA**T**L	CMQVWPAYG**N**	C4
*Setaria*	*Setaria italica*	PFQGLKSTA(**R/G**)L	CMQVWP(A/I)EG(**N/G)**	C4
*Miscanthus*	*Miscanthus x giganteus*	PFQGLKSTA**S**L	CMQVWPAYG**N**	C4
*Panicum*	*Panicum hallii*	PFQGLKSAA**G**L	CMQVWPTEN**N**	C4
*Zea*	*Zea mays*	PFQGLKSTA**S**L	CMQVWPAYG**N**	C4
*Oryza*	*Oryza barthii*	PFQGLKSTA**G**(L/M)	CMQVWPIEG**I**	C3
	*Oryza brachyantha*	PFQGLKSTA**G**M	CMQVWPIEG**I**	C3
	*Oryza glaberrima*	PFQGLKSTA**G**(L/M)	CMQVWPIEG**I**	C3
	*Oryza glumipatula*	PFQGLKSTA**G**(L/M)	CMQVWPIEG**I**	C3
	*Oryza meridionalis*	PFQGLKSTA**G**(L/M)	CMQVWPIEG**I**	C3
	*Oryza nivara*	PFQGLKSTA**G**(L/M)	CMQVWPIEG**I**	C3
	*Oryza punctata*	PFQGLKSTA(**G/D**)(L/M)	CMQVWP(V/I)(D/E)G(K)**I**	C3
	*Oryza rufipogon*	PFQGLKSTA**G**(L/M)	CMQVWPIEG**I**	C3
	*Oryza sativa*	PFQGLKSTA**G**(L/M)	CMQVWP(V/I)(D/E)G(K)**I**	C3
*Brachypodium*	*Brachypodium distachyon*	PFQGLKSTA**G**L	(S/C)MQVWPIEG**I**	C3
*Secale*	*Secale cereale*	PFQGLKSTA**G**L	CMQVWPIEG**I**	C3
*Leersia*	*Leersia perrieri*	PFQGLKSTA**G**(L/M)	CMQVWPIEG**I**	C3
*Bromus*	*Bromus catharticus*	PFQGLKSTA**G**L	CMQVWPIEG**I**	C3
*Phleum*	*Phleum pratense*	PFQGLKSTA**G**L	CMQVWPIEG**I**	C3
*Aegilops*	*Aegilops bicornis*	PFQGLKST(A/G)**G**L	CMQVWPIEG**I**	C3
	*Aegilops longissima*	PFQGLKSTA**G**L	CMQVWPIEG**I**	C3
	*Aegilops searsii*	PFQGLKSTA**G**L	CMQVWPIEG**I**	C3
	*Aegilops sharonensis*	PFQGLKSTA**G**L	CMQVWPIEG**I**	C3
	*Aegilops speltoides*	PFQGLKSTA**G**L	CMQVWPIEG**I**	C3
	*Aegilops tauschii*	PFQGLKSTA**G**L	CMQVWPIEG**I**	C3
*Triticum*	*Triticum aestivum*	PFQGLKSTA**G**L	CMQVWPIEG**I**	C3
	*Triticum dicoccoides*	PFQGLKSTA**G**L	CMQVWPIEG**I**	C3
	*Triticum timopheevii*	PFQGLKSTA**G**L	CMQVWPIEG**I**	C3
	*Triticum turgidum subsp. durum*	PFQGLKSTD**G**L	CMQVWPIEG**I**	C3
	*Triticum urartu*	PFQGLKST(A/T)**G**(L/M)	CMQVWPIEG**I**	C3
*Elytrigia*	*Thinopyrum intermedium*	PFQGLKSTA**G**L	CMQVWPIEG**I**	C3
*Avena*	*Avena agadiriana*	PFQGLKSTA**G**L	CMQVWPIEG**I**	C3
	*Avena clauda*	PFQGLKSTA**G**L	CMQVWPIEG**I**	C3
	*Avena maroccana*	PFQGLKSTA**G**L	CMQVWPIEG**I**	C3
	*Avena sativa*	PFQGLKSTA**G**L	CMQVWPIEG**I**	C3
	*Avena sterilis subsp. ludoviciana*	PFQGLKSTA**G**L	CMQVWPIEG**I**	C3
	*Avena strigosa*	PFQGLKSTA**G**L	CMQVWPIEG**I**	C3

a This region is located in the 16^th^-25^th^ residue of OsRbcS1.

b This region is located in the 47^th^-56^th^ residue of OsRbcS1.

c P-type represents the type of photosynthesis.

#### 3.3.3 Phosphoenolpyruvate carboxylase (PEPC)

There were no significant differences in gene structure or conserved domains observed between C_4_ and C_4_-orthologous PEPC genes/proteins, with both containing 10 exons in genes and 32 conserved domains in proteins (**Figure 2C**). The glycine residue (G) at position 842 of ZmPEPC4 was found to be specific in the C_4_ species of Gramineae (Rangan et al., 2016). Multiple alignments of the PEPC sequences of 65 species downloaded from the NCBI, UniProt, and Esembl Plants databases (**Table 5**) revealed that this G residue was relatively conserved in the PEPCs of C_3_ species, except soybean. Distinctly, at this site, A was conserved in dicotyledons, the *Flaveria* lineage with the C_3_-C_4_ intermediate photosynthetic type, and the unicellular C_4_ lineage *Hydrilla*, whereas G was conserved in monocotyledonous plants, especially all C_4_ grass species. A previous study showed that mutations in G could enhance tolerance to malic acid (Rangan et al., 2016); therefore, the mutation of A842G may be critical for the function of the C_4_ photosynthetic PEPC.

**Table 5:** Amino acid (9^th^ position, bold) for C_4_-specific PEPC.

Class	Family	Species	Amino acid with flanking region^a^	P-type^b^
Musci	*Funariaceae*	*Physcomitrella patens*	AKGDPRIA(**A/E/Q**)L	C3
dicots	*Amborellaceae*	*Amborella trichopoda*	AKGDPGIA**A**L	C3
	*Apiaceae*	*Daucus carota*	AKGDPGIA(**A/E**)L	C3
	*Asteraceae*	*Helianthus annuus*	AKGDPGIA**A**L	C3
	*Brassicaceae*	*Arabidopsis thaliana*	AKGDPGIA**A**L	C3
		*Arabidopsis halleri*	AKGDPGIA(**A/T**)L	C3
		*Brassica napus*	AKGDPGIA**A**L	C3
		*Brassica rapa*	AKGDPGIA**A**L	C3
	*Bromeliaceae*	*Ananas comosus*	AKGDPGIA**A**L	C3
		*Neoregelia ampullacea*	XXGDPGIA**A**L	C3
	*Chenopodiaceae*	*Beta vulgaris*	AKGDPGIA**A**L	C3
	*Cucurbitaceae*	*Cucumis sativus*	AKGDPGIA**A**L	C3
	*Euphorbiaceae*	*Manihot esculenta*	AKGDPGIA**A**L	C3
	*Hydrocharitaceae*	*Flaveria trinervia*	AKG(D/N)PGIA**A**L	C4
		*Flaveria pringlei*	AKGDPGIA**A**L	C3
		*Hydrilla verticillata*	AKGDP(G/V)IA**A**M	Facultative C4
	*Leguminosae*	*Vigna angularis*	AKGNPGIA(**A/V**)L	C3
		*Medicago truncatula*	AKGDPGIA**A**L	C3
		*lupinus angustifolius*	AKGDPGIA(**T/A**)L	C3
		*Glycine max*	AKGDPKIA(**A/G**)L	C3
		*Trifolium pratense*	AKGDPGIAAL	C3
		*Vigna radiata*	AKG(N/D)P(G/E)IA**A**L	C3
		*Phaseolus vulgaris*	AKGDP(K/G)I(G/A)**A**L	C3
	*Malvaceae*	*Gossypium raimondii*	AKGDPGIA**A**L	C3
		*Corchorus capsularis*	AKGDPGIA**A**L	C3
	*Musaceae*	*Musa acuminata*	AKGDPGIA**A**I	C3
	*Palmae*	*Elaeis guineensis*	AKGNPGIA**A**L	C3
	*Rosaceae*	*Prunus persica*	AKGNPGIA**A**L	C3
	*Salicaceae*	*Populus trichocarpa*	AKGDPGIA**A**L	C3
	*Sapindaceae*	*x Mokara* cv. *‘Yellow’*	SKGNSGIA**A**L	C3
	*Solanaceae*	*Nicotiana attenuata*	AKG(N/D)P(G/S)IA**A**L	C3
		*Solanum tuberosum*	AKGDPGIA**A**L	C3
		*Solanum lycopersicum*	AKGDPGIA**A**L	C3
	*Sterculiaceae*	*Theobroma cacao*	AKGDPGIA**A**L	C3
monocots	*Cyperaceae*	*Cyperus esculentus*	AKGDPGIA**A**L	C3
	*Orchidaceae*	*Dendrobium officinale*	TKGSSGIA**A**L	C3
		*Epidendrum stamfordianum*	TKGSPGIA**A**L	C3
		*Leptotes bicolor*	(A/T)KG(D/S)PGIA(**T/A**)L	C3
		*Microcoelia aphylla*	(A/T)(K/R)G(N/D)PGIA**A**L	C3
		*Microcoelia exilis*	A(K/Q)GDPGIA**A**L	C3
		*Phalaenopsis amabilis*	AKGDPGIA**A**L	C3
		*Phalaenopsis equestris*	AKGDPGIA**A**L	C3
		*Tillandsia usneoides*	AKGDPGIA**A**L	C3
		*Vanilla planifolia*	AKGNPGIA**S**L	C3
	*Poaceae*	*Aegilops tauschii*	AKGDPGIA**A**L	C3
		*Brachypodium distachyon*	AKGDPGIA**A**L	C3
		*Echinochloa crus-galli*	AKGDPGIVG^a^F	C4
		*Echinochloa glabrescens*	AKGDPGIA**A**L	C4
		*Zea luxurians*	AKGDPGIAG^a^L	C4
		*Hordeum vulgare*	AKGNPGIA**A**L	C3
		*Hordeum vulgare subsp. vulgare*	AKGNPGIA**A**L	C3
		*Hordeum vulgare subsp. spontaneum*	AKGNPGIA**A**L	C3
		*Oryza sativa subsp. indica*	AKGDPGIA**A**L	C3
		*Cenchrus americanus*	AKADPIIAG^a^L	C4
		*Saccharum officinarum*	AKGDPGIAG^a^L	C4
		*Saccharum spontaneum*	AKGDPGIA(G^a^/A)L	C4
		*Setaria italica*	AKG(N/D)P(T/G)IA(S/E/G^a^)L	C4
		*Sorghum bicolor*	AKG(D/N)PGIA(A/G^a^)(V/L)	C4
		*Triticum aestivum*	AKGDPGIA**A**L	C3
		*Triticum dicoccoides*	AKGDPRIA**A**L	C3
		*Triticum urartu*	AKG(D/N)PGIA**A**L	C3
		*Triticum monococcum*	AKGNPGIA**A**L	C3
		*Zea may*	AKG(N/D)PGIA(G^a^/A)(L/V)	C4
		*Zea mays subsp. mexicana*	AKGDPGIAG^a^L	C4
		*Zea mays subsp. parviglumis*	AKGDPGIAG^a^L	C4

a This region is located in the 834th-843th residue of ZmPEPC4.

b P-type represents the type of photosynthesis.

#### 3.3.4 NADP-dependent malic enzyme (NADP-ME)

No significant differences in gene structure or conserved domains were observed between C_4_ and C_4_-orthologous ME genes/proteins in C_4_ and C_3_ species, with both containing 20 exons (**Figure 2D**) encoding proteins with 591 to 648 aa (**Table S5**). Although differences in multiple amino acid residues were found between the C_4_ and C_4_-orthologous ME protein sequences in gramineous crops, such as C234V, A260S, R299K, and Q506E on OsME4, there was no evidence that these changes could impact enzyme kinetics. These variations could help to quickly distinguish C_4_ photosynthetic gene copies from non-photosynthetic gene copies in gramineous crops.

#### 3.3.5 Malate dehydrogenase (MDH)

In terms of gene structure, both C_4_-homologous MDHs and C_4_ MDHs contain 14 exons (**Figure 2E**). Except for the SiMDH protein with 493 aa, the lengths of the other MDHs range from 431 to 434 aa (**Table S6**). The differences in conserved domains between C_4_ and C_4_-orthologous MDHs were mainly concentrated in the N-terminal non-conserved regions. Differences in multiple amino acid residues were also found between the C_4_ MDH and C_4_-orthologous MDH protein sequences, such as D197E, A167V, and V267M.

#### 3.3.6 Pyruvate, orthophosphate dikinase (PPDK)

C_4_ and C_4_-orthologous PPDKs had no significant differences in gene structure or conserved domains, with both containing 19 exons (**Figure 2F**) encoding proteins of 936 to 948 aa in length. Similar to C_4_ *PPDK*, *PPDK* genes of C_3_ crops had special double promoter structures. There were differences in multiple amino acid residues between the C_4_ and C_4_-orthologous PPDK protein sequences, such as (T/S)92A, D95E, Q160A, and Q279E on OsPPDK1, with Q160A being present in highly conserved regions, but the effect of these differences on PPDK function needs to be confirmed.

### 3.4 Tissue-specific expression of these C_4_ and C_4_-orthologous genes

To compare the expression characteristics of these genes in C_3_ and C_4_ species, RNA-seq data from four tissues (leaf, root, spike, and shoot) of the five species were used. As FPKM values from different species could not be directly compared, the tissue expression characteristics of genes were determined based on inter-tissue standardization, and the original FPKM values are listed in the heatmap (**Figure 3**).

#### 3.4.1 β-CA

The *β-CA* genes in different branches showed great differences in tissue-specific expression. *β-CA1* genes were expressed in various tissues with high expression levels in leaves, roots, and spikes, while *β-CA2* genes had low expression levels in the four tissues and did not show significant differences between C_3_ and C_4_ crops. Unlike the relatively low expression levels of *β-CA1* and *β-CA2*, *β-CA3* exhibited ultrahigh expression levels in the leaves of both C_3_ and C_4_ crops, which might accelerate the diffusion of inorganic carbon to the carboxylase (Rubisco, PEPC) site and increase the CO_2_ fixation rate by increasing the concentration of inorganic carbon around the carboxylase (**Figure 3A**). The high expression of C_4_-orthologous *β-CA3* genes in C_3_ crop leaves suggested that they play an indispensable role in maintaining photosynthesis stability.

#### 3.4.2 RbcS

The *RbcS* genes of both C_3_ and C_4_ crops showed consistent tissue-specific expression patterns, and all were highly expressed in leaves (**Figure 3B**). Different *RbcS* gene copies of the same species showed different expression levels. For example, the expression levels of the two copies in the fifth homologous group (*TaRbcS7* and *TaRbcS8*) in wheat were much higher than those in the second homologous group (*TaRbcS4*, *TaRbcS5*, and *TaRbcS6*), and *SiRbcS5* in foxtail millet showed preferential expression. This indicated that the nonprimary gene copies may be functionally redundant or have a complementary function.

#### 3.4.3 PEPC

Each group of *PEPC* genes had a clear distinction in the specificity of tissue expression, while *PEPC* genes clustered in the same branch showed similar expression patterns in C_3_ and C_4_ crops (**Figure 3C**). For example, *PEPC1s* and *PEPC3s* were highly expressed in the spike and root, while *PEPC2s* were highly expressed only in the spike, and *PEPC4s* and *PEPC5s* were specifically expressed in the leaf. The current view is that non-photosynthetic PEPC copies are widely present in C_3_ and C_4_ species and are expressed in a variety of tissues to perform multiple functions (Aubry et al., 2011; Williams et al., 2012). The non-photosynthetic function of non-C_4_ *PEPC* copies not only replenishes tricarboxylic acid (TCA) cycle carbon skeletons, which is the traditional view of non-photosynthetic PEPC function, but also plays a nonnegligible role in seed formation and germination, fruit ripening, maintenance of cell ion balance, regulation of stomatal opening and provision of respiratory substrates for symbiotic nitrogen-fixing bacteroids of root nodules (Latzko and Kelly, 1983; Lepiniec et al., 2003). Based on sequence characteristics and expression patterns, the functions of these non-photosynthetic *PEPCs* were predicted. *PEPC1* and *PEPC3* may play a very important role in seed germination and the interaction of root microbes, and *PEPC5* may contribute to maintaining the pH environment and stomatal opening of leaf cells (O’Leary et al., 2011). Although *PEPC4* in C_3_ crops also showed a certain degree of leaf-specific expression, its expression level was much lower than that of C_4_ *PEPC4* in C_4_ species, which suggested that improving the expression level of *PEPC4* in C_3_ crops might be the first step for PEPC transformation.

#### 3.4.4 NADP-ME

*ME* gene copies within each group exhibited similar tissue expression specificity. *ME1* gene copies were expressed at very low levels in all tissues of all species (**Figure 3D**). Most *ME* gene copies in the second and third groups were specifically expressed in roots, while some gene copies from maize, sorghum, and rice (*ZmME2*, *OsME2*, and *SbME3*) were also highly expressed in leaves and spikes. Interestingly, *ME* gene copies in the fourth group exhibited different tissue expression characteristics; those from C_3_ species were specifically expressed in spikes (*OsME4* and *TaME4*), while those from C_4_ species were specifically expressed either in leaves or spikes, with *SiME4*, *ZmME4*, and *SbME4* being exclusively expressed in leaves and *ZmME5*, *ZmME6*, *SbME5*, and *SbME6* being highly expressed in spikes. *ME4* was suggested as the C_4_ photosynthetic orthologous group by evolutionary relationship analysis and subcellular localization (**Table 2**, **Table S5**). Therefore, the specific expression of *ME4* in leaves is the key to the C_4_ photosynthetic transformation of C_3_ ME.

#### 3.4.5 MDH

For NAD-dependent cytoplasmic MDHs, only *MDH1* genes encoding proteins of approximately 330 aa in length with a molecular weight of approximately 35 kDa could be expressed normally, while the other MDH gene copies (*MDH2*, *MDH10*, and *MDH11*) were not expressed or weakly expressed in various tissues of each species (**Figure 3E**, **Table S6**). Studies based on Chinese cabbage and alfalfa have confirmed that cytosolic MDH can affect grain development and response to salt stress (Luo et al., 2004; Jia and Zhang, 2009). High expression of *MDH1* in spikes and roots indicated that the cytoplasmic MDH of gramineous crops may have similar features. Seventeen NAD-dependent plastid *MDH* genes were highly expressed in three tissues: MDH5s and *OsMDH4* in the leaf; *OsMDH16*, *ZmMDH4*, *SbMDH4*, and *ZmMDH18* in the root; and *TaMDH4* and *TaMDH15* in the spike. Previous studies have shown that transgenic *Arabidopsis*, which lacks plastid NAD-dependent *MDH*, exhibits a variety of negative effects, such as reduced chlorophyll content, decreased photosynthetic rate, disordered chloroplast ultrastructure and undevelopable seeds (van Roermund et al., 2004). Plastid NAD-MDH gene copies with leaf-specific expression were essential for plant photosynthesis, while those with spike-specific expression affected grain development. In addition, the higher number of leaf-specific cytoplasmic *MDH* gene copies in C_4_ species may ensure the stability of dimorphic chloroplasts in MCs and BSCs. There were two highly conserved copies of peroxisome *MDH* in each species: *MDH6* was specifically expressed in the root, while *MDH7* was specifically expressed in the spike. The root growth of *Arabidopsis* lacking peroxisome *MDH* was severely hindered due to incomplete β-oxidation. Sequence alignment showed that At2g22780 had higher homology with *MDH6*, so *MDH6* might play an important role in the β-oxidation process (Pracharoenwattana et al., 2007). In addition, quantitative proteomic analysis of peroxisomes in *Arabidopsis thaliana* confirmed that another peroxisome MDH (At5g09660) is essential for photorespiration, suggesting that *MDH7* might perform similar functions in gramineous crops, which is consistent with the high expression level of *MDH7* in C_3_ crops (Eubel et al., 2008). Mitochondrial *MDH* was mainly expressed in the spike and root. As a traditional TCA cycle enzyme in plants, its high expression in the spike and root may be related to the active energy metabolism of these two tissues. As a key gene of the C_4_ photosynthetic pathway, NADP-dependent chloroplast *MDH4* was highly expressed in the leaves of C_4_ species but not in C_3_ species, which indicated that increasing the expression level of *MDH4* in C_3_ species is essential for C_4_ photosynthetic transformation.

#### 3.4.6 PPDK

Because of the existence of a dual-promoter structure, two *PPDK* gene copies in C_4_ crops encoded three PPDK isoforms, and there were two and seven PPDK isoforms in rice and wheat, respectively (**Table S7**). Both chloroplast mRNAs (*PPDK1.1*) in C_3_ and C_4_ crops were highly expressed in leaves, while the cytoplasmic mRNAs were highly expressed in spikes (**Figure 3F**). The chloroplast *PPDK* gene copies in C_3_ crops were expressed specifically in leaves, similar to the *PPDK* gene copies in C_4_ crops, but their expression levels were much lower. This indicated that the high expression of chloroplast *PPDK* was key to the conversion of C_3_ to C_4_.

### 3.5 Comparison of the promoter sequences of C_4_ and C_4_-orthologous gene copies

The completeness of the C_4_ photosynthetic pathway involves the improved expression levels and specific-tissue/cell expression of the key pathway genes, and the noncoding region of the gene harbours the information for its gene expression and cell-specific expression. Therefore, we compared the noncoding regions in the upstream promoter regions of C_4_ orthologues in C_3_ crops and C_4_ genes in C_4_ crops.

Differences in the -1000 to -2000 region upstream of the initiation codon were found in the promoter region between C_4_-orthologous *β-CA* in C_3_ crops and C_4_ *β-CA* in C_4_ crops (**Figure S4**). The prediction of cis-regulatory elements found that C_4_-orthologous *β-CA* genes had more CAAT-box elements (C(C/A)AAT) and more light-responsive elements in the -1000 to -2000 region than C_3_ *β-CA* genes, which suggests that the C_4_-orthologous *β-CA* could be easily recruited into the C_4_ photosynthetic pathway.

There was a significant difference in the region approximately 300 bp upstream of the initiation codon between the C_4_ *RbcS* genes in C_4_ crops and the C_4_-orthologous *RbcS* genes in C_3_ crops. A conserved domain cluster consisting of 3 conserved motifs (motif 6+motif 1+motif 7) was found to be specific to C_4_ *RbcS* genes, and further prediction revealed that this region contained multiple specific elements, such as abscisic acid-responsive (ABRE) and MeJA responsive (CGTCA-motif) elements (**Figure S5**), as well as a CAT-box, which controls meristem-specific expression. This may be one of the key regulatory elements for the high expression of C_4_ *RbcS* in BSCs.

More elements interacting with MYB transcription factors were observed in the promoter regions of C_4_ *PEPC* genes of C_4_ crops than in those of C_4_-orthologous *PEPC* genes of C_3_ crops, which were enriched in three regions of the initiation codon, -100 to -600 bp, -1000 to -1400 bp and -1600 to -2000 bp (**Figure S6**). The multiple MYB binding sites might be induced by drought and light, which indicated that MYB transcription factors may participate in the regulation of C_4_ *PEPC* expression levels under stress conditions.

Large differences were observed in the promoter regions of C_4_/C_4_-orthologous *ME* and *MDH* genes from different C_3_ and C_4_ crops, and no common motifs or cis-acting elements were found in the promoter regions of C_4_ *ME* and *MDH* genes. In general, multiple abiotic elements involved in the abiotic stress response, hormone response, and light response were found in the promoter regions of all *ME* and *MDH* genes, which suggested that they may be regulated in multiple ways.

There was a difference in the expression levels of the C_4_ *PPDK* and C_4_-orthologous *PPDK* genes. However, no significant difference in the promoter region was found between the C_4_ and C_4_-orthologous *PPDK* genes. This result suggested that the leaf-specific expression of *PPDK* might be regulated in other ways.

### 3.6 The expression synergy of C_4_ genes and C_4_ orthologues in leaves

**Figure 4:**
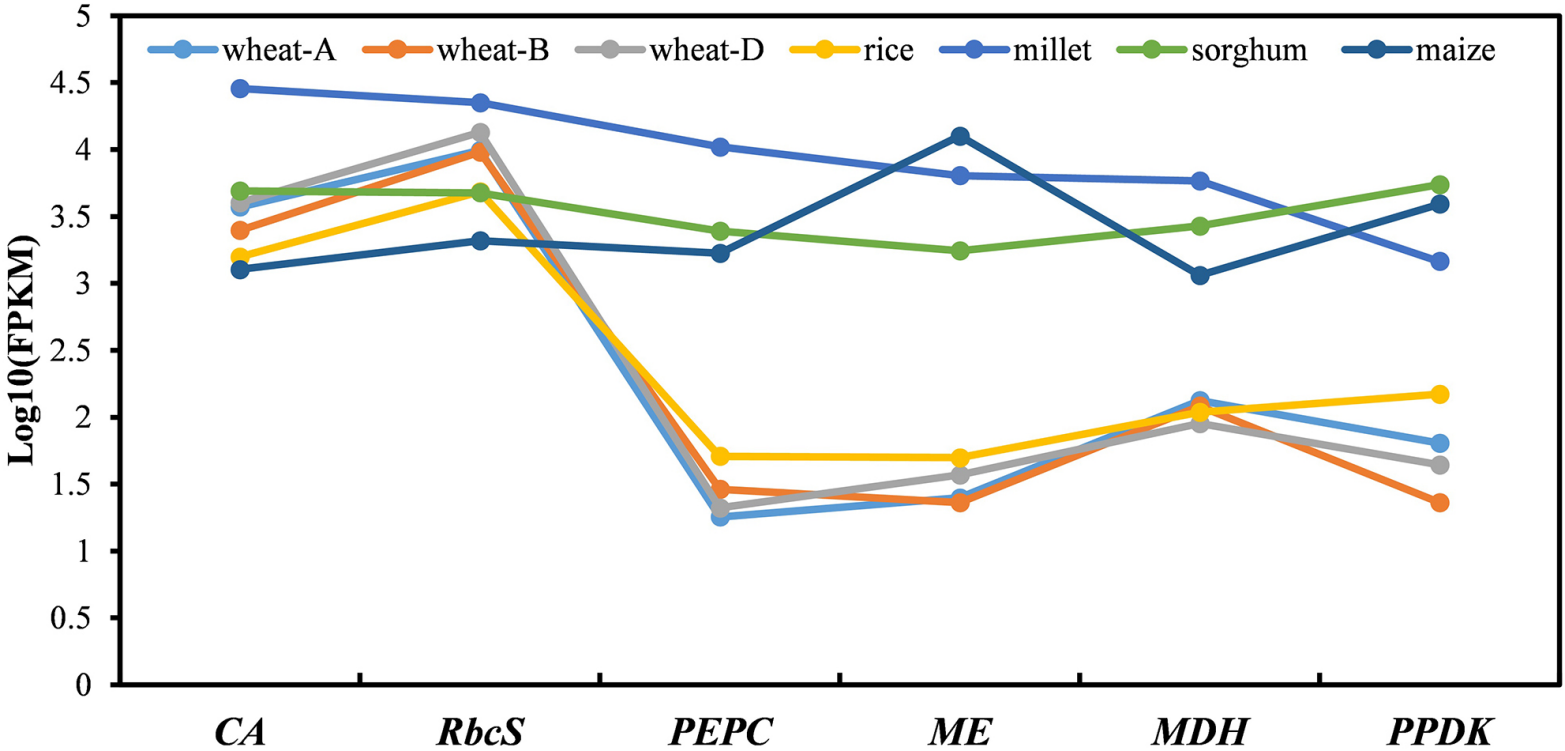
The expression synergy of the C_4_ genes and C_4_ orthologues in leaves of C_4_ and C_3_ species. The different coloured lines/dots represent the C_4_/C_4_-orthologues encoding six key enzymes in different gramineous crops. Wheat-A, wheat-B, and wheat-D represent three copies of the different subgenomes of bread wheat. Millet represents foxtail millet. The genes presented include *β-CA3*, *PEPC4*, *ME4*, *MDH3*, and *PPDK1.1* orthologous groups, and the *RbcS* genes with the highest expression in each species. Because no *PEPC4* orthologue was found in rice, the nearest paralogue, *OsPEPC3*, was used instead.

The C_4_ photosynthetic pathway is a complete and stable system, and its smooth operation requires synergy in the expression levels of multiple genes involved in this pathway. Therefore, we explored the difference in the expression synergy in leaves between C_4_ genes in C_4_ crops and C_4_ orthologues in C_3_ crops. As no C_4_-orthologous gene copy was found in rice, a closely orthologous gene (*OsPEPC3*) was used instead. All six C_4_ genes were highly expressed cooperatively in the leaves of C_4_ crops, while for the C_4_-orthologous genes, only *RbcS* and *β-CA* in C_3_ crops had similar expression levels in leaves to those of C_4_ genes in the leaves of C_4_ crops, and the remaining genes, *PEPC*, *MDH*, *ME*, and *PPDK*, were all expressed at much lower levels in leaves of C_3_ crops (**Figure 4**). This indicated that although C_4_-orthologous genes existed in C_3_ crops, their expression could not be well coordinated to form the full C_4_ photosynthetic pathway. Therefore, increasing and coordinating the expression levels of these C_4_-orthologous genes are of great importance for the activation of the C_4_ pathway in C_3_ crops.

## 4 Discussion

### 4.1 Key enzymes in the C_4_ photosynthetic pathway of C_3_ and C_4_ Gramineae crops

In this study, almost all homologous genes encoding the six key enzymes of the C_4_ photosynthetic pathway were identified in C_3_ crops, consistent with the finding that all genes required for the C_4_ pathway are present and expressed in C_3_ species (Aubry et al., 2011; Williams et al., 2012). Based on genome-wide analysis, more gene copies were found than previously reported, which is highly advantageous. Moreover, the annotations of *PPDK* were revised based on our in-depth analysis of *PPDK* genes.

Almost all the C_4_-homologous genes in C_3_ crops of wheat and rice were identified based on the analysis of phylogenetic relationships, tissue expression characteristics, and subcellular localization. In addition to their C_4_ photosynthetic gene copies, at least three non-photosynthetic paralogous copies were found in the *β-CA*, *PEPC*, *ME*, and *MDH* gene families, and similar gene structure and protein sequence characteristics were observed among the orthologous genes from different crops, which indicated that the non-photosynthetic homologous genes were relatively conserved in C_3_ and C_4_ crops during the evolution of the C_4_ photosynthetic pathway. Consistent with previous studies, four gene lineages of independent origin from those of C_4_ grass crops, *β-CA3*, *PEPC4*, *ME4*, and *MDH3*, were found to have been recruited to the C_4_ photosynthetic pathway, confirming the identity of the gene lineages during C_4_ evolution, and these gene copies in C_3_ crops are important candidates for gene manipulation (Christin et al., 2013; Moreno-Villena et al., 2017). Moreover, the positive effects of gene duplication on the expansion of these gene families were confirmed, as 83.33% of *RbcS* genes and 73.53% of *β-CA* genes were associated with tandem duplication events, with more duplicated genes in the branches with C_4_ photosynthetic gene copies. As previously reported, gene duplication was the first step in the evolution of C_4_ photosynthesis (Sage et al., 2012).

### 4.2 The difference between the C_4_-ortholous genes in C_3_ crops and the C_4_ genes in C_4_ crops

With the evolution of the C_4_ photosynthetic pathway, the ancestral C_3_ genes underwent modification of the regulatory region or mutation of the coding region, resulting in the adjustment of the intracellular position of key enzymes, obvious kinetic characteristics, and cell-specific expression patterns (Ludwig, 2013; Ludwig, 2016). These changes were also found among the six gene families between the C_3_ and C_4_ Gramineae crops investigated in this study; for example, ZmCA3, SiCA3, and SbCA3 were all cytoplasmic, while OsCA3 and TaCA3s were located in chloroplasts. Based on the alignment of the conserved domains of C_4_ β-CA and C_4_-orthologous β-CA, C_4_ β-CA was found to be accumulated in the cytoplasm due to the deletion of a conserved motif at the N-terminus, which led to loss of the chloroplast transit peptide.

Previous studies showed that the encoded enzymes likely varied in their kinetic properties in addition to their leaf and cell specificities. The enzymatic kinetic changes in the C_4_ photosynthetic pathway mainly occurred in the two major carboxylases PEPC and Rubisco (Bläsing et al., 2002; Kapralov et al., 2011). The PEPCs from C_3_ and C_4_ *Flaveria* species exhibit different kinetic properties in terms of substrate saturation and response to activators and inhibitors, while the PEPC of C_3_-C_4_ *Flaveria* species showed features between those of the C_3_ and C_4_ congeners, suggesting that the C_4_ characteristics were gradually obtained during the evolution process (Engelmann et al., 2003). It has been confirmed that the serine residue (serine 774 of the C_4_ *F. trinervia* PEPC) at the C-terminus of the C_4_ PEPC protein determines the difference in substrate affinity (Bläsing et al., 2002); similarly, this feature was found in the present study. In addition, based on large-scale multiple sequence alignment, two ((T/S)24G and N56I on OsRbcS1) and one (G842A on ZmPEPC4) amino acid mutations in C_4_ RbcS and PEPC were found to be highly specific to C_4_ Gramineae crops and were located in highly conserved regions of these proteins. The 8 amino acid residues at positions 52-59 of OsRbcS1 constitute a protein binding site, and the significant variation at position 56 may have a critical impact on the function of Rubisco. In addition, specific amino acid substitutions have been associated with C_4_ functionality. In the C_4_ PEPC isoform of maize, Increased tolerance to feedback inhibition by malate involves specific G884 (Glycine) in C_4_-isoforms (Paulus et al., 2013). The G842A mutation in ZmPEPC4 may have a similar effect on PEPC enzyme kinetics. In summary, the discovery of these mutation sites may provide a basis for the interpretation of the novel kinetic characteristics of key C_4_ carboxylases.

### 4.3 The promoter regions and the expression characteristics of C_4_ genes and C_4_-orthologous genes in C_4_/C_3_ crops

Different C_4_ orthologues in C_3_ crops exhibited different expression characteristics. All C_4_ genes in C_4_ crops were highly expressed in leaves. *RbcS* and *β-CA* in C_3_ and C_4_ crops showed similar expression levels, while the C_4_ orthologues of *PEPC*, *ME*, *MDH*, and *PPDK* exhibited much lower expression in the leaves of C_3_ crops than that observed for the C_4_ genes in the leaves of C_4_ crops. C_4_-orthologous *RbcS* in C_3_ crops was highly expressed in MCs, and C_4_ *RbcS* in C_4_ species was highly expressed in BSCs. In this study, we observed differences in tissue expression and subcellular localization of gene copies of the same lineage between C_3_ and C_4_ crops, and high levels of expression in leaves and localization to specific organelles were confirmed to be key for recruitment to the C_4_ photosynthetic pathway, which is consistent with previous reports (Ludwig, 2016; Moreno-Villena et al., 2017). Multiple elements related to hormone response-related and meristem-specific expression were found 300 bp upstream of the C_4_ *RbcS* initiation codon, which may directly regulate the specific expression of C4 *RbcS* in BSCs. Previous promoter deletion experiments on the maize RbcS promoter region confirmed that deletion of the -211 to +434 region resulted in the accumulation of RbcS in BSCs, which is consistent with our results (Purcell et al., 1995). However, regional differences exist in the three representative C_4_ crops, confirming that high expression of C_4_ *RbcS* in BSCs is regulated similarly.

In addition to the discovery that the promoter region regulated the cell expression targeting of the C_4_ gene, regions that may regulate PEPC expression levels were also found. The C_4_ PEPC promoter regions had more cis-regulatory elements related to MYB transcription factors, including multiple light- and drought-inducible MYB binding sites. This indicated that regulation by MYB transcription factors may play a positive role in the recruitment of PEPC to the C_4_ pathway. Studies have shown that the 0.6 kb upstream sequence of the maize PEPC promoter, which corresponds to region 2, can bind to proteins to regulate MC-specific expression of PEPC (Bläsing et al., 2002), and a mesophyll enhancement module (MEM1) at the distal end (-1566 to -2141) of the C_4_ PEPC promoter of *F. bidentis*, which corresponds to region 3, was predicted in this study (Akyildiz et al., 2007). In general, the Gramineae C_4_ species and *F. bidentis* C_4_ species seem to have similar regulatory patterns for C_4_ *PEPC*.

Unfortunately, no additional elements that may regulate the expression levels of C_4_ MDH, ME, or PPDK were found in their promoter regions. The manner in which the promoter regions of these genes are regulated in Gramineae C_4_ species may be different, with the regulation possibly being more determined by the process of DNA methylation and trans-acting factors.

### 4.4 The synergistic expression of C_4_ and C_4_-orthologous genes in leaves of C_4_/C_3_ crops

As a complete system, the expression of individual genes involved in the C_4_ photosynthetic pathway must be well coordinated to form a complete and stable metabolic pathway. Our analysis showed that the expression levels of multiple C_4_ orthologues, including *PEPC*, *PPDK*, *MDH*, and *ME*, were much lower and not well coordinated in the leaves of C_3_ crops; therefore, improving and coordinating their expression might be a strategy for activating the C_4_ pathway in C_3_ crops.

**Figure 5:**
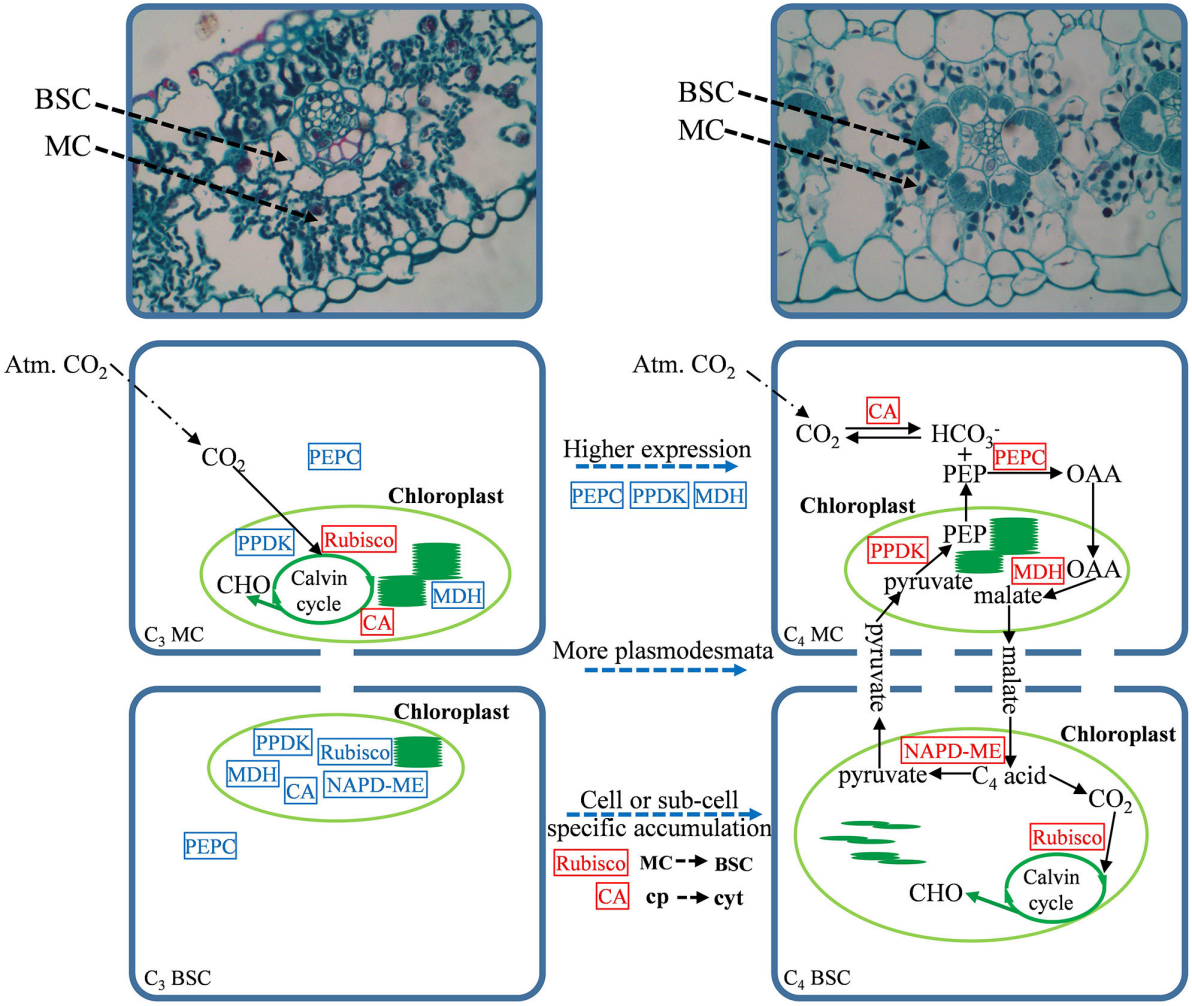
Schema of C_3_ photosynthesis and NADP-malic enzyme (NADP-ME)-type C_4_ photosynthesis in gramineous crops. From top to bottom are the anatomical structure and photosynthetic model. The dotted line in the middle marks the necessary steps for the photosynthetic pathway to evolve from C_3_ to C_4_. Cp is the chloroplast, cyt is the cytoplasm, and the red box and blue box represent high/low expression levels, respectively.

Based on the analysis in this study, the necessary steps for the conversion of the C_4_-homologous photosynthetic pathway to the C_4_ photosynthetic pathway in the currently examined C_3_ crops were proposed (**Figure 5**). The acquisition of the C_4_ photosynthetic pathway involves not only the improvement of the expression level of the key genes in the pathway but also a variety of changes, including cell-specific expression patterns, adjustments in the intracellular location of the enzymes, and their kinetic characteristics. Therefore, altering the expression level of one or two genes is not sufficiently effective for the establishment of the entire C_4_ system. In addition, studies based on maize, rice, and *Cleome* species have shown that some regulatory information determining cell-specific expression already exists in the ancestral C_3_ gene, and the expression characteristics and compartmentalization modification observed in the C_4_ species are regulated by at least one trans-acting factor or transcription factor (Aubry et al., 2011). Therefore, a wider range of in-depth comparisons of C_3_ crops and their related C_4_ crops is necessary for the transformation of the C_4_ photosynthetic pathway in C_3_ crops. In addition, great progress has been made in improving Rubisco’s CO_2_ specificity by genetic manipulation and significantly improving the photosynthetic efficiency of C_3_ crops (Martinavila et al., 2020); the redesign of the bypass pathway based on photorespiration also greatly improved the photosynthetic efficiency of rice (Shen et al., 2019), which may also be an effective way to improve the photosynthetic efficiency of wheat and C_3_ crops. Although in this study, a large number of key regions and amino acid variations that may affect the transformation of C_4_-homologous genes to C_4_ genes were observed in the C_4_-orthologous genes of C_3_ crops, more experiments should be performed to verify these findings in the future.

## 5 Conclusion

In this study, the genes encoding the 6 key enzymes of the C_4_ photosynthetic pathway in five important gramineous crops were systematically identified and characterized. The C_4_ functional gene copies were distinguished from the non-photosynthetic functional gene copies based on phylogenetic relationships, subcellular localization, and expression characteristics. Two mutations ((T/S)24G and N56I on OsRbcS1) of RbcS and one mutation (G842A on ZmPEPC4) of PEPC were found to be specific to the C_4_ crops, which may affect the enzymatic kinetics of major carboxylases. Possible cis-acting elements regulating the specific expression of C_4_ genes were found in their promoter regions. The expression of most C_4_ orthologues in the leaves of C_3_ crops was much lower and not well coordinated, and the necessary steps for C_4_ photosynthetic transformation of C_3_ crops were proposed. The results of this study will help progress research into the C_4_ photosynthetic transformation of important C_3_ species, such as rice and wheat.

## Data availability statement

The datasets presented in this study can be found in online repositories. The names of the repository/repositories and accession number(s) can be found in the article/**Supplementary Material**.

## Author contributions

Y-GH, LC, YY, and MP conceived and designed the original research. YY, LC, ZZ, and SL performed the experiments and analyzed the data; QL and CC assisted the data analysis; LC and YY wrote the manuscript; MP and Y-GH complemented the writing. All authors contributed to the article and approved the submitted version.
