# Supplementary material for: Genome-wide identification and comparative analyses of key genes involved in C4 photosynthesis in five main gramineous crops
Source: Front Plant Sci. 2023 Mar 13;14:1134170. doi: 10.3389/fpls.2023.1134170 (PMC10040670; doi:10.3389/fpls.2023.1134170)
Supplement: Supplementary file 1 [file DataSheet_1.docx]

**Supplementary Material**

Genome-Wide Identification and Comparative analyses of Key Genes involved in C_4_ Photosynthesis in Five Main Gramineous Crops

Liang Chen^1,†^, Yang Yang^2,†^, Zhangchen Zhao^1^, Shan Lu^1^, Qiumei Lu^1^, Chunge Cui^1^, Martin Parry^3^,Yin-Gang Hu^1, 4*^

^1^ State Key Laboratory of Crop Stress Biology for Arid Areas, College of Agronomy, Northwest A&F University, Yangling, Shaanxi, China

^2^ College of Agriculture, Shannxi Agricultural University (Institute of Crop Sciences), Taiyuan, Shannxi, China

^3^ Lancaster Environment Centre, Lancaster University, Lancaster, UK

^4^ Institute of Water Saving Agriculture in Arid Regions of China, Northwest A&F University, Yangling, Shaanxi, China

^†^These authors contributed equally to this work

^*^ Correspondence:

Yin-Gang Hu

huyingang@nwsuaf.edu.cn

**Supplementary File**

SupFile 1. All_query_protein_sequences_for_BLASTP.fa

**Supplementary Figures:**

Figure legends:

**Fig S1 Phylogenetic relationships between six gene families involved in C_4_ photosynthetic pathway from common wheat (Ta), maize (Zm), foxtail millet (Si), rice (Os), sorghum (Sb), and *Arabidopsis* (At) based on their amino acid sequences.**

Multiple sequence alignment and phylogenetic tree construction were performed using MUSCLE and MEGA 6.0 (Neighbor-Joining method), respectively. Scale bars indicate the number of amino acid substitutions per site. Genes with Bold blue font represent C_4_-type copies in C_4_ species. A: β-CA; B: RbcS; C: PEPC; D: NADP-ME; E: MDH; F: PPDK.

**Fig S2 Variations in the conserved domains between C_4_ and C_4_-homologous β-CA in C_4_ and C_3_ crops.**

The conserved domains of β-CA s were predicted and displayed by MEME and TBtools, respectively. The chloroplast transit peptides were predicted by chlorop v1.1

**Fig S3 Variations in the conserved domains between C_4_ and C_4_-homologous RbcS** **in C_4_ and C_3_ crops.**

The conserved domains of RbcSs were predicted and displayed by MEME and TBtools, respectively. The protein binding site was predicted on PredictProtein (<https://predictprotein.org>).

**Fig S4 The characteristics in the promoter region of C_4_ and C_4_ homologous *β-CA* genes in C_4_ and C_3_ crops.**

The conserved domains were predicted and displayed by MEME and TBtools, respectively. The cis-regulatory elements were predicted by PlantCARE (<http://bioinformatics.psb.ugent.be/webtools/plantcare/html/>).

**Fig S5 The characteristics in the promoter region of C_4_ and C_4_ homologous *RbcS* genes** **in C_4_ and C_3_ crops.**

The conserved domains were predicted and displayed by MEME and TBtools, respectively. The cis-regulatory elements were predicted by PlantCARE (<http://bioinformatics.psb.ugent.be/webtools/plantcare/html/>).

**Fig S6 The characteristics in the promoter region of C_4_ and C_4_ homologous *PEPC* genes in C_4_ and C_3_ crops.**

The cis-regulatory elements were predicted by PlantCARE (<http://bioinformatics.psb.ugent.be/webtools/plantcare/html/>).

**Supplementary Tables:**

**Table S1 Information on transcriptome data downloaded from public databases**

| Species | Stage | Tissue | SRA/DEO ID |
| --- | --- | --- | --- |
| Sorghum | Shoots from 9 d seedings | Shoot | SRP029353 |
| Sorghum | 20 days after sowing | Leaf | SRP008469 |
| Sorghum | Shoots from 9 d seedings | Root | SRP029353 |
| Sorghum | the milk stage of grain development | Spike | SRP029353 |
| Maize | three leaf stage | Internode | SRP010680 |
| Maize | three leaf stage | Leaf | SRP010680 |
| Maize | three leaf stage | Root | SRP010680 |
| Maize | three leaf stage | Embryos | SRP010680 |
| Foxtail Millet | 40 days after germination | Stem | SRP011401 |
| Foxtail Millet | 40 days after germination | Leaf | SRP011401 |
| Foxtail Millet | 40 days after germination | Root | SRP011401 |
| Foxtail Millet | 40 days after germination | Spica | SRP011401 |
| Common Wheat | 30% spike | Peduncle | PRJEB25639 |
| Common Wheat | 30% spike | Leaf | PRJEB25639 |
| Common Wheat | 30% spike | Root | PRJEB25639 |
| Common Wheat | 30% spike | Spike | PRJEB25639 |
| Rice | 14 days after germination | Shoot | SRP028766 |
| Rice | 14 days after germination | Leaf | SRP049212 |
| Rice | 14 days after germination | Root | SRP028766 |
| Rice | 7 days after flowering | Panicle | DRP000391 |

**Table S2 Characteristics of the putative β-CA proteins in five Gramineous crops**

| **Species** | **Gene** | **Sequence ID** | **Location** | **AA**  **Length** | **Molecular**  **Weight** | **Subcellular Location** | **High Expression**  **Tissue** | **Gene Type (non-C_4_, C_4_, C_4_-homologous)** | **Duplication event** |
| --- | --- | --- | --- | --- | --- | --- | --- | --- | --- |
| Millet | *SiCA 1* | *SETIT_030616mg* | Chr2:33801767-33804057 | 269 | 30254.39 | chloroplast | shoot leaf root spike | non-C_4_ |  |
| Millet | *SiCA 2* | *SETIT_002669mg* | Chr5:30350305-30354010 | 250 | 27786.30 | chloroplast | spike | non-C_4_ | TD1 |
| Millet | *SiCA3* | *SETIT_003882mg* | Chr5:30333130-30336956 | 336 | 36644.11 | cytoplasm | leaf | C_4_ | TD1 |
| Millet | *SiCA4* | *SETIT_002140mg* | Chr5:30341562-30344631 | 242 | 26378.56 | chloroplast | root | non-C_4_ | TD1 |
| Sorghum | *SbCA1* | *SORBI_3002G230100* | Chr2:62133898-62137600 | 306 | 33497.88 | chloroplast | root leaf shoot | non-C_4_ |  |
| Sorghum | *SbCA2* | *SORBI_3003G234600* | Chr3:57323907-57327962 | 256 | 28448.1 | chloroplast | low | non-C_4_ | TD2 |
| Sorghum | *SbCA3* | *SORBI_3003G234200* | Chr3:57297987-57310502 | 448 | 48986.93 | cytoplasm | leaf | C_4_ | TD2 |
| Sorghum | *SbCA4* | *SORBI_3003G234500* | Chr3:57318984-57321883 | 174 | 19259.36 | chloroplast | root | non-C_4_ | TD2 |
| Sorghum | *SbCA8* | *SORBI_3003G234400* | Chr3:57311562-57316959 | 204 | 22418.85 | chloroplast | leaf shot | non-C_4_ | TD2 |
| Maize | *ZmCA1a* | *Zm00001d020764* | Chr7:131557949-131561931 | 250 | 27040.70 | chloroplast | root leaf | non-C_4_ |  |
| Maize | *ZmCA1b* | *Zm00001d005920* | Chr2:192762349-192765881 | 304 | 33438.77 | chloroplast | leaf | non-C_4_ |  |
| Maize | *ZmCA3* | *Zm00001d044099* | Chr3:219075378-219080211 | 200 | 21980.31 | cytoplasm | leaf | C_4_ | TD3 |
| Maize | *ZmCA4* | *Zm00001d044095* | Chr3:219031166-219034215 | 209 | 23017.60 | chloroplast | leaf low | non-C_4_ | TD3 |
| Maize | *ZmCA9* | *Zm00001d044096* | Chr3:219038137-219043675 | 239 | 26383.39 | chloroplast | leaf | non-C_4_ | TD3 |
| Maize | *ZmCA10* | *Zm00001d011454* | Chr8:150927227-150934087 | 245 | 26535.77 | chloroplast | leaf | non-C_4_ |  |
| Rice | *OsCA1* | *Os09t0464000* | Chr9:17567637-17571539 | 306 | 33768.21 | chloroplast | leaf spike | non-C_4_ |  |
| Rice | *OsCA2* | *Os01t0640000* | Chr1:25708316-25716827 | 237 | 26989.28 | chloroplast | low | non-C_4_ | TD4 |
| Rice | *OsCA3* | *Os01t0639900* | Chr1:25696671-25705077 | 272 | 29117.42 | chloroplast | leaf spike | C_4_-homologous | TD4 |
| Wheat | *TaCA1-5A* | *TraesCS5A02G245700* | Chr5A:458968240-458972760 | 307 | 33748.95 | chloroplast | leaf spike root | non-C_4_ |  |
| Wheat | *TaCA1-5B* | *TraesCS5B02G243100* | Chr5B:423226447-423229963 | 307 | 33613.86 | chloroplast | root leaf spike | non-C_4_ |  |
| Wheat | *TaCA1-5D* | *TraesCS5D02G252400* | Chr5D:358011403-358015146 | 289 | 31859.84 | chloroplast | spike root leaf | non-C_4_ |  |
| Wheat | *TaCA2-3A* | *TraesCS3A02G230100* | Chr3A:430521723-430524839 | 250 | 28190.74 | chloroplast | low | non-C_4_ | TD5 |
| Wheat | *TaCA2-3B* | *TraesCS3B02G259400* | Chr3B:417368034-417371221 | 250 | 28268.77 | chloroplast | low | non-C_4_ | TD6 |
| Wheat | *TaCA2-3D* | *TraesCS3D02G223200* | Chr3D:304031533-304034891 | 250 | 28283.87 | chloroplast | low | non-C_4_ | TD7 |
| Wheat | *TaCA3-3A* | *TraesCS3A02G230000* | Chr3A:430330494-430337814 | 259 | 28076.34 | chloroplast | leaf shoot spike | C_4_-homologous | TD5 |
| Wheat | *TaCA3-3B* | *TraesCS3B02G259300* | Chr3B:417258446-417265886 | 258 | 27963.18 | chloroplast | leaf shoot spike | C_4_-homologous | TD6 |
| Wheat | *TaCA3-3D* | *TraesCS3D02G223300* | Chr3D:304327703-304335024 | 258 | 27977.21 | chloroplast | leaf shoot spike | C_4_-homologous | TD7 |
| Wheat | *TaCA5-7A* | *TraesCS7A02G454300* | Chr7A:649071954-649076024 | 246 | 26969.12 | chloroplast | leaf | non-C_4_ | TD8 |
| Wheat | *TaCA5-7B* | *TraesCS7B02G354800* | Chr7B:613176304-613179047 | 202 | 22409.80 | chloroplast | root leaf | non-C_4_ | TD9 |
| Wheat | *TaCA5-7D* | *TraesCS7D02G443400* | Chr7D:563526506-563529389 | 202 | 22407.77 | chloroplast | leaf | non-C_4_ | TD10 |
| Wheat | *TaCA6-7A* | *TraesCS7A02G454500* | Chr7A:649184449-649194668 | 213 | 23899.39 | chloroplast | low | non-C_4_ | TD8 |
| Wheat | *TaCA6-7B* | *TraesCS7B02G354900* | Chr7B:613253108-613256883 | 244 | 27135.17 | chloroplast | root | non-C_4_ | TD9 |
| Wheat | *TaCA6-7D* | *TraesCS7D02G443500* | Chr7D:563662671-563666267 | 198 | 21985.28 | chloroplast | root | non-C_4_ | TD10 |
| Wheat | *TaCA7-7A* | *TraesCS7A02G454400* | Chr7A:649150422-649154246 | 239 | 26504.36 | chloroplast | root | non-C_4_ | TD8 |

TD represents the tandem duplication event and the number represents the different group, respectively.

**Table S3 Characteristics of the putative RbcS proteins in five Gramineous crops**

| **Species** | **Gene** | **Sequence ID** | **Location** | **AA**  **Length** | **Molecular**  **Weight** | **Subcellular Location** | **High Expression Tissue** | **Gene Type (non-C_4_, C_4_, C_4_-homologous)** | **Duplication**  **event** |
| --- | --- | --- | --- | --- | --- | --- | --- | --- | --- |
| Millet | *SiRbcS1* | SETIT_023461mg | Chr3:37910629-37911778 | 168aa | 19181.13 | Chloroplast | leaf | C4 | TD1 |
| Millet | *SiRbcS2* | SETIT_023437mg | Chr3:37959935-37961153 | 173aa | 19466.51 | Chloroplast | leaf | C4 | TD1 |
| Millet | *SiRbcS3* | SETIT_023462mg | Chr3:38100098-38101091 | 168aa | 19181.13 | Chloroplast | leaf | C4 |  |
| Millet | *SiRbcS4* | SETIT_023466mg | Chr3:24069118-24070242 | 169aa | 19084.04 | Chloroplast | leaf | C4 | TD2 |
| Millet | *SiRbcS5* | SETIT_023465mg | Chr3:24102022-24103068 | 169aa | 19082.08 | Chloroplast | leaf | C4 | TD2 |
| Sorghum | *SbRbcS1* | SORBI_3005G042000 | Chr5:3876057-3877378 | 169aa | 19058.87 | Chloroplast | leaf | C4 |  |
| Maize | *ZmRbcS1* | Zm00001d004894 | Chr2:147269761-147270804 | 170aa | 19090.94 | Chloroplast | leaf | C4 |  |
| Maize | *ZmRbcS2* | Zm00001d052595 | Chr4:194257728-194258862 | 170aa | 19150.99 | Chloroplast | leaf | C4 |  |
| Rice | *OsRbcS1* | Os12t0274700 | Chr12:10080505-10081588 | 175aa | 19646.68 | Chloroplast | leaf | non-C4 |  |
| Rice | *OsRbcS2* | Os12t0291100 | Chr12:11262587-11263539 | 175aa | 19630.66 | Chloroplast | leaf | non-C4 | TD3 |
| Rice | *OsRbcS3* | Os12t0291400 | Chr12:11277247-11278306 | 175aa | 19498.5 | Chloroplast | leaf | non-C4 | TD3 |
| Rice | *OsRbcS4* | Os12t0292400 | Chr12:11320409-11322505 | 175aa | 19660.65 | Chloroplast | leaf | non-C4 |  |
| Wheat | *TaRbcS1-2A* | TraesCS2A02G066700 | Chr2AS:30071558-30072776 | 174aa | 19374.33 | Chloroplast | leaf | non-C4 | TD4 |
| Wheat | *TaRbcS2-2A* | TraesCS2A02G066800 | Chr2AS:30125890-30127338 | 175aa | 19505.46 | Chloroplast | leaf | non-C4 | TD4 |
| Wheat | *TaRbcS3-2A* | TraesCS2A02G066900 | Chr2AS:30020633-30021500 | 192aa | 21124.24 | Chloroplast | leaf | non-C4 | TD4 |
| Wheat | *TaRbcS4-2A* | TraesCS2A02G067100 | Chr2AS:30090306-30091759 | 175aa | 19481.48 | Chloroplast | leaf | non-C4 | TD4 |
| Wheat | *TaRbcS5-2A* | TraesCS2A02G067200 | Chr2AS:30085405-30086867 | 175aa | 19440.42 | Chloroplast | leaf | non-C4 | TD4 |
| Wheat | *TaRbcS6-2A* | TraesCS2A02G067300 | Chr2AS:30079116-30080690 | 175aa | 19463.44 | Chloroplast | leaf | non-C4 | TD4 |
| Wheat | *TaRbcS1-2B* | TraesCS2B02G078900 | Chr2BL:44360475-44361961 | 182aa | 20499.75 | Chloroplast | leaf | non-C4 | TD5 |
| Wheat | *TaRbcS2-2B* | TraesCS2B02G079100 | Chr2BL:44190500-44192003 | 174aa | 19443.44 | Chloroplast | leaf | non-C4 | TD5 |
| Wheat | *TaRbcS3-2B* | TraesCS2B02G079200 | Chr2BL:44346180-44347892 | 175aa | 19491.44 | Chloroplast | leaf | non-C4 | TD5 |
| Wheat | *TaRbcS4-2B* | TraesCS2B02G079300 | Chr2BL:44310557-44311687 | 174aa | 19374.33 | Chloroplast | leaf | non-C4 | TD5 |
| Wheat | *TaRbcS5-2B* | TraesCS2B02G079400 | Chr2BL:44285559-44286975 | 175aa | 19463.44 | Chloroplast | leaf | non-C4 | TD5 |
| Wheat | *TaRbcS6-2B* | TraesCS2B02G079500 | Chr2BL:44272600-44274689 | 193aa | 21244.55 | Chloroplast | leaf | non-C4 | TD5 |
| Wheat | *TaRbcS1-2D* | TraesCS2D02G065100 | Chr2DL:27887631-27889099 | 174aa | 19433.4 | Chloroplast | leaf | non-C4 | TD6 |
| Wheat | *TaRbcS2-2D* | TraesCS2D02G065200 | Chr2DL:27859563-27860987 | 175aa | 19461.41 | Chloroplast | leaf | non-C4 | TD6 |
| Wheat | *TaRbcS3-2D* | TraesCS2D02G065300 | Chr2DL:27883022-27884222 | 174aa | 19388.36 | Chloroplast | leaf | non-C4 | TD6 |
| Wheat | *TaRbcS4-2D* | TraesCS2D02G065400 | Chr2DL:27853295-27854235 | 175aa | 19477.47 | Chloroplast | leaf | non-C4 | TD6 |
| Wheat | *TaRbcS5-2D* | TraesCS2D02G065500 | Chr2DL:27901255-27902757 | 165aa | 18266.04 | Chloroplast | leaf | non-C4 | TD6 |
| Wheat | *TaRbcS6-2D* | TraesCS2D02G065600 | Chr2DL:27891136-27892278 | 175aa | 19463.44 | Chloroplast | leaf | non-C4 | TD6 |
| Wheat | *TaRbcS7-5A* | TraesCS5A02G165400 | Chr5AL:353988332-353989702 | 175aa | 19490.51 | Chloroplast | leaf | non-C4 | TD7 |
| Wheat | *TaRbcS7-5B* | TraesCS5B02G162600 | Chr5BL:299847320-299848476 | 175aa | 19490.51 | Chloroplast | leaf | non-C4 | TD8 |
| Wheat | *TaRbcS7-5D* | TraesCS5D02G169600 | Chr5DL:266085742-266086801 | 175aa | 19490.51 | Chloroplast | leaf | non-C4 | TD9 |
| Wheat | *TaRbcS8-5A* | TraesCS5A02G165700 | Chr5AL:354314588-354315579 | 175aa | 19490.51 | Chloroplast | leaf | non-C4 | TD7 |
| Wheat | *TaRbcS8-5B* | TraesCS5B02G162800 | Chr5BL:300097036-300098231 | 175aa | 19490.51 | Chloroplast | leaf | non-C4 | TD8 |
| Wheat | *TaRbcS8-5D* | TraesCS5D02G169900 | Chr5DL:266325112-266326079 | 175aa | 19490.51 | Chloroplast | leaf | non-C4 | TD9 |

TD represents the tandem duplication event and the number represents the different group, respectively.

**Table S4 Characteristics of the putative PEPC proteins in five Gramineous crops**

| **Groups** | **Species** | **Gene** | **Sequence ID** | **Location** | **AA**  **Length** | **Molecular**  **Weight** | **Subcellular Location** | **High Expression Tissue** | **C_4_-orthologous** |
| --- | --- | --- | --- | --- | --- | --- | --- | --- | --- |
| Class I | Sorghum | *SbPEPC1* | SORBI_3002G167000 | Chr2:52122128-52127601 | 967aa | 110176.3 | Chloroplast | root | non-C4 |
|  | Sorghum | *SbPEPC6* | SORBI_3007G106500 | Chr7:38044938-38050518 | 964aa | 110057.2 | Chloroplast | root | non-C4 |
|  | Millet | *SiPEPC1* | SETIT_028826mg | Chr2:26199104-26204493 | 967aa | 110202.3 | Chloroplast | root | non-C4 |
|  | Maize | *ZmPEPC1* | Zm00001d020057 | Chr7:89268035-89273487 | 978aa | 111317.6 | Chloroplast | root | non-C4 |
|  | Rice | *OsPEPC1* | Os09t0315700 | Chr9:8692296-8697399 | 975aa | 110474.3 | Chloroplast | root | non-C4 |
|  | Rice | *OsPEPC6* | Os08t0366000 | Chr8:16964711-16970563 | 964aa | 110056.1 | Chloroplast | spike | non-C4 |
|  | Wheat | *TaPEPC1-5A* | TraesCS5A02G181800 | Chr5AL:381365734-381371670 | 973aa | 110221.1 | Chloroplast | root | non-C4 |
|  | Wheat | *TaPEPC1-5B* | TraesCS5B02G179800 | Chr5BL:327603933-327609868 | 973aa | 110186 | Chloroplast | root | non-C4 |
|  | Wheat | *TaPEPC1-5D* | TraesCS5D02G186200 | Chr5DL:288179911-288185588 | 973aa | 110186 | Chloroplast | root | non-C4 |
| Class II | Sorghum | *SbPEPC2* | SORBI_3003G301800 | Chr3:63252659-63260241 | 966aa | 110106.1 | Chloroplast | spike | non-C4 |
|  | Millet | *SiPEPC2* | SETIT_000184mg | Chr5:37379789-37386482 | 969aa | 110440.3 | Chloroplast | spike | non-C4 |
|  | Maize | *ZmPEPC2* | Zm00001d012702 | Chr8:178935189-178935486 | 966aa | 110160.2 | Chloroplast | spike | non-C4 |
|  | Rice | *OsPEPC2* | Os01t0758300 | Chr1:31859334-31865031 | 924aa | 105314.5 | Chloroplast | spike | non-C4 |
|  | Wheat | *TaPEPC2-3A* | TraesCS3A02G306700 | Chr3AL:544389833-544396960 | 967aa | 110084.2 | Chloroplast | spike | non-C4 |
|  | Wheat | *TaPEPC2-3B* | TraesCS3B02G329800 | Chr3BL:532930223-532936865 | 967aa | 110061.1 | Chloroplast | spike | non-C4 |
|  | Wheat | *TaPEPC2-3D* | TraesCS3D02G295200 | Chr3DL:407230389-407237181 | 967aa | 110129.1 | Chloroplast | spike | non-C4 |
| Class III | Sorghum | *SbPEPC3* | SORBI_3004G106900 | Chr4:10201753-10208601 | 960aa | 109365.8 | Chloroplast | root, spike | non-C4 |
|  | Millet | *SiPEPC3* | SETIT_016228mg | Chr1:1784597-1791242 | 965aa | 109819.3 | Chloroplast | root, spike | non-C4 |
|  | Maize | *ZmPEPC3* | Zm00001d053453 | Chr4:232245967-232253821 | 896aa | 101830 | Chloroplast | root, spike | non-C4 |
|  | Rice | *OsPEPC3* | Os02t0244700 | Chr2:8172421-8184167 | 968aa | 109932.6 | Chloroplast | root, spike | non-C4 |
|  | Wheat | *TaPEPC3-6A* | TraesCS6A02G195600 | Chr6AS:282127418-282148245 | 969aa | 110091.6 | Chloroplast | root, spike | non-C4 |
|  | Wheat | *TaPEPC3-6B* | TraesCS6B02G223100 | Chr6BS:331033701-331052537 | 969aa | 110054.6 | Chloroplast | root, spike | non-C4 |
|  | Wheat | *TaPEPC3-6D* | TraesCS6D02G183200 | Chr6DS:219781439-219795962 | 969aa | 110147.7 | Chloroplast | root, spike | non-C4 |
| Class IV  (C4-type) | Sorghum | *SbPEPC4* | SORBI_3010G160700 | Chr10:47244445-47253575 | 1028aa | 115731.7 | Chloroplast | leaf | C4 |
|  | Millet | *SiPEPC4* | SETIT_005789mg | Chr4:28034710-28043142 | 964aa | 109982.7 | Chloroplast | leaf | C4 |
|  | Maize | *ZmPEPC4* | Zm00001d046170 | Chr9:68851094-68856482 | 970aa | 109341 | Chloroplast | leaf | C4 |
|  | Wheat | *TaPEPC4-7A* | TraesCS7A02G345400 | Chr7AL:507757190-507763146 | 919aa | 104542.4 | Chloroplast | leaf | C4-orthologous |
|  | Wheat | *TaPEPC4-7B* | TraesCS7B02G237900 | Chr7BL:443292417-443299286 | 892aa | 101122.3 | Chloroplast | leaf | C4-orthologous |
|  | Wheat | *TaPEPC4-7D* | TraesCS7D02G333900 | Chr7DL:425502517-425509832 | 968aa | 109614.9 | Chloroplast | leaf | C4-orthologous |
| Class V | Millet | *SiPEPC5* | SETIT_000160mg | Chr5:13060669-13065012 | 1015aa | 113528.5 | Chloroplast | leaf | non-C4 |
|  | Rice | *OsPEPC5* | Os01t0208700 | Chr1:5899561-5909560 | 1014aa | 114197.3 | Chloroplast | leaf | non-C4 |
|  | Wheat | *TaPEPC5-3A* | TraesCS3A02G134200 | Chr3AS:111550279-111554136 | 1004aa | 113502.3 | Chloroplast | leaf | non-C4 |
|  | Wheat | *TaPEPC5-3B* | TraesCS3B02G168000 | Chr3BS:168679370-168683332 | 1003aa | 113471.3 | Chloroplast | leaf | non-C4 |
|  | Wheat | *TaPEPC5-3D* | TraesCS3D02G150500 | Chr3DS:115018932-115022824 | 1004aa | 113488.3 | Chloroplast | leaf | non-C4 |

**Table S5 Characteristics of the putative NADP-ME proteins in five Gramineous crops**

| **Groups** | **Species** | **Gene** | **Sequence ID** | **Location** | **AA**  **Length** | **Molecular**  **Weight** | **Subcellular Location** | **High Expression Tissue** | **C_4_-orthologous** |
| --- | --- | --- | --- | --- | --- | --- | --- | --- | --- |
| Group I | Sorghum | *SbME1* | SORBI_3003G292400 | Chr3:62488685-62493225 | 593aa | 64781.16 | cytoplasm | spike | non-C4 |
|  | Millet | *SiME1* | SETIT_000808mg | Chr5:36544632-36549204 | 580aa | 63707.99 | cytoplasm | root | non-C4 |
|  | Maize | *ZmME1* | Zm00001d012764 | Chr8:180332662-180336615 | 497aa | 54762.95 | cytoplasm | spike | non-C4 |
|  | Rice | *OsME1* | Os01t0743500 | Chr1:31076571-31081251 | 585aa | 64269.75 | cytoplasm | spike(low) | non-C4 |
|  | Wheat | *TaME1-3A* | TraesCS3A02G275600 | Chr3AL:505221882-505226419 | 568aa | 62332.34 | cytoplasm | root(low) | non-C4 |
|  | Wheat | *TaME1-3B* | TraesCS3B02G309300 | Chr3BL:497709387-497714785 | 621aa | 68233.2 | cytoplasm | root(low) | non-C4 |
|  | Wheat | *TaME1-3D* | TraesCS3D02G275500 | Chr3DL:381990604-381995438 | 690aa | 75738.95 | chloroplast | root(low) | non-C4 |
| Group II | Sorghum | *SbME2* | SORBI_3003G280900 | Chr3:61585619-61591072 | 593aa | 65551.83 | cytoplasm | root, leaf | non-C4 |
|  | Millet | *SiME2* | SETIT_000774mg | Chr5:35586907-35591980 | 593aa | 65450.67 | cytoplasm | root, leaf | non-C4 |
|  | Maize | *ZmME2* | Zm00001d043601 | Chr3:204885528-204890476 | 664aa | 72863.15 | chloroplast | root, leaf | non-C4 |
|  | Rice | *OsME2* | Os01t0723400 | Chr1:30166020-30171659 | 642aa | 71500.63 | cytoplasm | leaf | non-C4 |
|  | Wheat | *TaME2-3A* | TraesCS3A02G285900 | Chr3AL:514310972-514316411 | 594aa | 65687.21 | cytoplasm | root | non-C4 |
|  | Wheat | *TaME2-3B* | TraesCS3B02G320200 | Chr3BL:519057048-519062660 | 638aa | 70389.7 | cytoplasm | root(low) | non-C4 |
|  | Wheat | *TaME2-3D* | TraesCS3D02G285700 | Chr3DL:394926749-394932625 | 661aa | 72907.27 | cytoplasm | root | non-C4 |
| Group III | Sorghum | *SbME3* | SORBI_3009G069600 | Chr9:8034359-8038174 | 570aa | 62738.64 | cytoplasm | stem | non-C4 |
|  | Millet | *SiME3* | SETIT_021600mg | Chr3:7278988-7282331 | 576aa | 63771.7 | cytoplasm | spike, root | non-C4 |
|  | Maize | *ZmME7* | Zm00001d010358 | Chr8:110966311-110969781 | 570aa | 62703.59 | cytoplasm | NA | non-C4 |
|  | Maize | *ZmME3* | Zm00001d037693 | Chr6:134213323-134216929 | 570aa | 62893.99 | cytoplasm | root | non-C4 |
|  | Rice | *OsME3* | Os05t0186300 | Chr5:5293558-5297417 | 570aa | 62934.86 | cytoplasm | spike | non-C4 |
|  | Wheat | *TaME3-1A* | TraesCS1A02G122500 | Chr1AS:138225347-138228929 | 570aa | 63125.06 | cytoplasm | root | non-C4 |
|  | Wheat | *TaME3-1B* | TraesCS1B02G141700 | Chr1BS:189321919-189326794 | 570aa | 63075.01 | cytoplasm | spike | non-C4 |
|  | Wheat | *TaME3-1D* | TraesCS1D02G123400 | Chr1DS:125067983-125071629 | 570aa | 63089.03 | cytoplasm | spike | non-C4 |
| Group IV  (C4-type) | Sorghum | *SbME4* | SORBI_3003G036200 | Chr3:3317184-3322427 | 636aa | 69377.63 | chloroplast | leaf | C4 |
|  | Sorghum | *SbME5* | SORBI_3003G036000 | Chr3:3299756-3305602 | 586aa | 64622.94 | cytoplasm | spike | C4-orthologous |
|  | Sorghum | *SbME6* | SORBI_3009G108700 | Chr9:43545770-43551420 | 652aa | 71535.71 | chloroplast | spike | C4-orthologous |
|  | Millet | *SiME4* | SETIT_000645mg | Chr5:11687298-11692722 | 639aa | 70037.89 | chloroplast | leaf | C4 |
|  | Maize | *ZmME4* | Zm00001eb121470 | Chr3:7276387-7281737 |  |  |  |  |  |
|  | Maize | *ZmME5* | Zm00001d037961 | Chr6:143609078-143614548 | 644aa | 70694.85 | chloroplast | leaf | C4-orthologous |
|  | Maize | *ZmME6* | Zm00001d037962 | Chr6:143657640-143663172 | 644aa | 70794.09 | chloroplast | spike | C4-orthologous |
|  | Rice | *OsME4* | Os01t0188400 | Chr1:4739271-4744472 | 639aa | 69865.84 | chloroplast | spike, root | C4-orthologous |
|  | Wheat | *TaME4-3A* | TraesCS3A02G108900 | Chr3AL:74781464-74787353 | 648aa | 70942.07 | chloroplast | spike, leaf | C4-orthologous |
|  | Wheat | *TaME4-3B* | TraesCS3B02G128000 | Chr3BS:106962508-106968100 | 591aa | 65203.5 | chloroplast | spike, leaf | C4-orthologous |
|  | Wheat | *TaME4-3D* | TraesCS3D02G110700 | Chr3DL:64370009-64375502 | 642aa | 70286.32 | chloroplast | spike, leaf | C4-orthologous |

**Table S6 Characteristics of the putative MDH proteins in Gramineous crops**

| **Groups** | **Species** | **Gene** | **Sequence ID** | **Location** | **AA**  **Length** | **Molecular**  **Weight** | **Subcellular Location** | **High Expression Tissue** | **C_4_-orthologous** |
| --- | --- | --- | --- | --- | --- | --- | --- | --- | --- |
| Class I-α  (NAD-type) | Sorghum | *SbMDH1* | SORBI_3001G219300 | Chr1:20443589-20448787 | 332aa | 35464.8 | cytoplasm | root, spike | non-C4 |
|  | Sorghum | *SbMDH2* | SORBI_3006G170800 | Chr6:52734301-52736982 | 354aa | 38653.38 | cytoplasm | stem(low) | non-C4 |
|  | Millet | *SiMDH1* | SETIT_036550mg | Chr9:16519097-16523865 | 332aa | 35483.78 | cytoplasm | leaf | non-C4 |
|  | Millet | *SiMDH2* | SETIT_010442mg | Chr7:26571867-26573596 | 358aa | 38998.73 | cytoplasm | stem(low) | non-C4 |
|  | Maize | *ZmMDH10* | Zm00001d014030 | Chr5:29404683-29410087 | 312aa | 33257.51 | cytoplasm | stem | non-C4 |
|  | Maize | *ZmMDH1* | Zm00001d032695 | Chr1:234902658-234908089 | 332aa | 35589.9 | cytoplasm | root, spike | non-C4 |
|  | Maize | *ZmMDH2* | Zm00001d002741 | Chr2:21195675-21197462 | 390aa | 42735.56 | cytoplasm | NA | non-C4 |
|  | Maize | *ZmMDH11* | Zm00001d019330 | Chr7:28121896-28123724 | 379aa | 41841.71 | cytoplasm | NA | non-C4 |
|  | Rice | *OsMDH1* | Os10t0478200 | Chr10:17913831-17917765 | 332aa | 35568.88 | cytoplasm | spike, root | non-C4 |
|  | Rice | *OsMDH2* | Os04t0551200 | Chr4:27605166-27608318 | 352aa | 38297.05 | cytoplasm | stem(low) | non-C4 |
|  | Wheat | *TaMDH1-1A* | TraesCS1A02G155200 | Chr1AL:272611172-272613645 | 328aa | 35074.46 | cytoplasm | spike, root | non-C4 |
|  | Wheat | *TaMDH1-1B* | TraesCS1B02G172400 | Chr1BL:307425225-307428664 | 333aa | 35485.88 | cytoplasm | spike, root | non-C4 |
|  | Wheat | *TaMDH1-1D* | TraesCS1D02G153900 | Chr1DL:214397868-214401005 | 333aa | 35475.84 | cytoplasm | spike, root | non-C4 |
| Class I-β  (NADP-type, C4) | Sorghum | *SbMDH14* | SORBI_3006G170800 | Chr7:60149510-60153216 | 429aa | 46455.06 | chloroplast | leaf | C_4_ |
|  | Sorghum | *SbMDH3* | SORBI_3007G166200 | Chr7:60144110-60147894 | 434aa | 46880.56 | chloroplast | leaf | C_4_ |
|  | Millet | *SiMDH13* | SETIT_029817mg | Chr2:15715146-15719068 | 452aa | 49380.29 | chloroplast | leaf | C_4_ |
|  | Millet | *SiMDH3* | SETIT_013632mg | Chr6:35757135-35760887 | 493aa | 52791.61 | chloroplast | leaf | C_4_ |
|  | Maize | *ZmMDH3* | Zm00001d031899 | Chr1:205992187-205996030 | 432aa | 46786.63 | chloroplast | leaf | C_4_ |
|  | Rice | *OsMDH3* | Os08t0562100 | Chr8:28141176-28144882 | 433aa | 47008.9 | chloroplast | leaf | C_4_-orthologous |
|  | Wheat | *TaMDH12-U* | TraesCSU02G127700 | ChrUn:109114737-109118860 | 389aa | 42185.36 | chloroplast | root | C_4_-orthologous |
|  | Wheat | *TaMDH12-5A* | TraesCS5A02G549900 | Chr5AL:703665977-703669406 | 389aa | 42185.36 | chloroplast | root | C_4_-orthologous |
|  | Wheat | TaMDH3-7B | TraesCS7B02G197000 | Chr7BL:339613762-339617788 | 431aa | 46682.5 | chloroplast | leaf | C_4_-orthologous |
|  | Wheat | TaMDH3-7D | TraesCS7D02G283900 | Chr7DS:296507630-296511690 | 431aa | 46814.67 | chloroplast | leaf | C_4_-orthologous |
|  | Wheat | TaMDH3-U | TraesCSU02G135100 | ChrUn:116430718-116434600 | 431aa | 46814.67 | chloroplast | leaf | C_4_-orthologous |
| Class II-β  (NAD-type) | Sorghum | *SbMDH5* | SORBI_3002G385700 | Chr2:74038065-74041790 | 388aa | 40528.38 | chloroplast | leaf | non-C4 |
|  | Millet | *SiMDH5* | SETIT_030117mg | Chr2:46369380-46370742 | 390aa | 40778.65 | chloroplast | leaf | non-C4 |
|  | Maize | *ZmMDH5* | Zm00001d022229 | Chr7:173196557-173197711 | 384aa | 40034.71 | chloroplast | leaf(low) | non-C4 |
|  | Rice | *OsMDH5* | Os07t0630800 | Chr7:26153825-26156006 | 404aa | 42221.7 | chloroplast | leaf(low) | non-C4 |
| Class III-α  (NAD-type) | Sorghum | *SbMDH6* | SORBI_3008G186200 | Chr8:62080570-62086083 | 446aa | 47099.71 | Peroxisome | root | non-C4 |
|  | Millet | *SiMDH6* | SETIT_022438mg | Chr3:50146670-50151253 | 363aa | 37885.91 | Peroxisome | root | non-C4 |
|  | Maize | *ZmMDH6* | Zm00001d041243 | Chr3:107817035-107822167 | 360aa | 37808.94 | Peroxisome | root | non-C4 |
|  | Rice | *OsMDH6* | Os12t0632700 | Chr12:27094649-27099336 | 356aa | 37385.37 | Peroxisome | root | non-C4 |
|  | Wheat | *TaMDH6-5A* | TraesCS5A02G014300 | Chr5AS:9650482-9654885 | 358aa | 37332.18 | Peroxisome | root | non-C4 |
|  | Wheat | *TaMDH6-5B* | TraesCS5B02G012400 | Chr5BS:12329987-12334657 | 358aa | 37256.05 | Peroxisome | root | non-C4 |
|  | Wheat | *TaMDH6-5D* | TraesCS5D02G019700 | Chr5DS:12591750-12596115 | 358aa | 37350.24 | Peroxisome | root | non-C4 |
| Class III-β  (NAD-type) | Sorghum | *SbMDH7* | SORBI_3001G073900 | Chr1:5624795-5634742 | 442aa | 46062.01 | Peroxisome | leaf | non-C4 |
|  | Millet | *SiMDH7* | SETIT_036365mg | Chr9:4283109-4286368 | 355aa | 37138.06 | Peroxisome | leaf | non-C4 |
|  | Maize | *ZmMDH7* | Zm00001d034241 | Chr1:287663868-287667586 | 358aa | 37463.3 | Peroxisome | leaf | non-C4 |
|  | Rice | *OsMDH7* | Os03t0773800 | Chr3:32086056-32089677 | 354aa | 37023.01 | Peroxisome | leaf | non-C4 |
|  | Wheat | *TaMDH7-5A* | TraesCS5A02G407700 | Chr5AL:598389423-598393155 | 377aa | 37262.05 | Peroxisome | leaf | non-C4 |
|  | Wheat | *TaMDH7-5B* | TraesCS5B02G412500 | Chr5BL:587471705-587475380 | 373aa | 37302.12 | Peroxisome | leaf | non-C4 |
|  | Wheat | *TaMDH-5D* | TraesCS5D02G417600 | Chr5DL:479784899-479788900 | 357aa | 37263.04 | Peroxisome | leaf | non-C4 |
| Class III-γ  (NAD-type) | Sorghum | *SbMDH8* | SORBI_3009G240700 | Chr9:57799754-57804273 | 340aa | 35347.77 | mitochondrion | spike | non-C4 |
|  | Millet | *SiMDH8* | SETIT_022574mg | Chr3:9555323-9559758 | 341aa | 35523.89 | mitochondrion | spike | non-C4 |
|  | Maize | *ZmMDH19* | Zm00001d009640 | Chr8:74045695-74049815 | 368aa | 38581.54 | mitochondrion | root | non-C4 |
|  | Maize | *ZmMDH8* | Zm00001d039089 | Chr6:169782693-169787359 | 340aa | 35285.69 | mitochondrion | root | non-C4 |
|  | Rice | *OsMDH8* | Os05t0574400 | Chr5:28617700-28621468 | 340aa | 35435.85 | mitochondrion | spike | non-C4 |
|  | Wheat | *TaMDH8-1A* | TraesCS1A02G412900 | Chr1AL:573060338-573065094 | 341aa | 35526.06 | mitochondrion | spike | non-C4 |
|  | Wheat | *TaMDH8-1B* | TraesCS1B02G443200 | Chr1BL:664297130-664302657 | 341aa | 35480.03 | mitochondrion | leaf | non-C4 |
|  | Wheat | *TaMDH8-1D* | TraesCS1D02G420500 | Chr1DL:476904707-476909192 | 341aa | 35496.03 | mitochondrion | spike | non-C4 |
|  | Sorghum | *SbMDH9* | SORBI_3003G238500 | Chr3:57798790-57804310 | 482aa | 51757.45 | mitochondrion | spike | non-C4 |
|  | Millet | *SiMDH9* | SETIT_002110mg | Chr5:30733947-30737280 | 340aa | 35479.85 | mitochondrion | spike | non-C4 |
|  | Maize | *ZmMDH9* | Zm00001d044042 | Chr3:217573164-217581039 | 340aa | 35640 | mitochondrion | spike | non-C4 |
|  | Rice | *OsMDH9* | Os01t0649100 | Chr1:26190754-26194403 | 340aa | 35460.89 | mitochondrion | spike | non-C4 |
|  | Wheat | *TaMDH9-3A* | TraesCS3A02G234800 | Chr3AL:438599373-438603027 | 340aa | 35463.85 | mitochondrion | spike | non-C4 |
|  | Wheat | *TaMDH9-3B* | TraesCS3B02G265000 | Chr3BL:423811858-423816588 | 340aa | 35452.83 | mitochondrion | spike | non-C4 |
|  | Wheat | *TaMDH9-3D* | TraesCS3D02G236200 | Chr3DL:326700697-326704621 | 340aa | 35466.86 | mitochondrion | spike | non-C4 |

**Table S7 Characteristics of the putative PPDK proteins in Gramineous crops**

| **Species** | **Gene** | **Sequence ID** | **Location** | **AA**  **Length** | **Molecular**  **Weight** | **Subcellular Location** | **High Expression Tissue** | **C_4_-orthologous** |
| --- | --- | --- | --- | --- | --- | --- | --- | --- |
| Sorghum | SbPPDK1.1 | SORBI_3009G132900.1 | Chr9:48726358-48738528 | 948aa | 102490.21 | chloroplast | leaf | C4 |
| Sorghum | SbPPDK1.2 | SORBI_3009G132900.2 | Chr9:48725978-48726400 | 882aa | 95885.6 | cytoplasm | NA | non-C_4_ |
| Sorghum | SbPPDK2 | SORBI_3001G326900 | Chr1:61372602-61380996 | 909aa | 99152.68 | cytoplasm | spike | non-C_4_ |
| Millet | SiPPDK1.1 | XP_004962130.1 | Chr3:21258962-21273297 | 945aa | 102424.94 | chloroplast | leaf | C4 |
| Millet | SiPPDK1.2 | SETIT_021174mg | Chr3:21266320-21273408 | 882aa | 95760.35 | cytoplasm | NA | non-C_4_ |
| Millet | SiPPDK2 | SETIT_034163mg | Chr4:41401484-41409136 | 893aa | 97184.45 | cytoplasm | spike | non-C_4_ |
| Maize | ZmPPDK1.1 | Zm00001d038163_P002 | Chr6:150024486-150035717 | 936aa | 101251 | chloroplast | leaf | C4 |
| Maize | ZmPPDK1.2 | Zm00001d038163_P003 | Chr6:150029624-150035717 | 882aa | 95806.65 | cytoplasm | NA | non-C_4_ |
| Maize | ZmPPDK2 | NP_001345328.1 | Chr8:109464175-109469697 | 883aa | 96076.14 | cytoplasm | spike | non-C_4_ |
| Rice | OsPPDK1.1 | Os05t0405000-01 | Chr5:19718538-19737605 | 947aa | 102787.88 | chloroplast | leaf | C4 |
| Rice | OsPPDK1.2 | Os05t0405000-02 | Chr5:19718538-19726410 | 882aa | 96229.44 | cytoplasm | spike | non-C_4_ |
| Wheat | TaPPDK1-1A.1 | TraesCS1A02G253400.1 | Chr1AL:445257710-445277310 | 939aa | 101861.56 | chloroplast | leaf | C_4_-orthologous |
| Wheat | TaPPDK1-1A.2 | TraesCS1A02G253400.3 | Chr1AL:445261054-445266932 | 665aa | 72220.53 | cytoplasm | spike | non-C_4_ |
| Wheat | TaPPDK2-1A | TraesCS1A02G253200.1 | Chr1AL:444768026-444776770 | 884aa | 96294.16 | cytoplasm | spike | non-C_4_ |
| Wheat | TaPPDK1-1B.1 | TraesCS1B02G264900.1 | Chr1BL:465683651-465701383 | 939aa | 101950.77 | chloroplast | leaf | C_4_-orthologous |
| Wheat | TaPPDK1-1B.2 | TraesCS1B02G264900.2 | Chr1BL:465692855-465701383 | 884aa | 96195.12 | cytoplasm | leaf | non-C_4_ |
| Wheat | TaPPDK1-1D.1 | TraesCS1D02G252900.1 | Chr1DL:345264118-345282757 | 939aa | 101845.56 | chloroplast | leaf | C_4_-orthologous |
| Wheat | TaPPDK1-1D.2 | TraesCS1D02G252900.2 | Chr1DL:345265872-345272206 | 638aa | 69594.28 | cytoplasm | root | non-C_4_ |
