# Supplementary figures and images for: Genome-wide identification and comparative analyses of key genes involved in C4 photosynthesis in five main gramineous crops
Source: Front Plant Sci. 2023 Mar 13;14:1134170. doi: 10.3389/fpls.2023.1134170 (PMC10040670; doi:10.3389/fpls.2023.1134170)

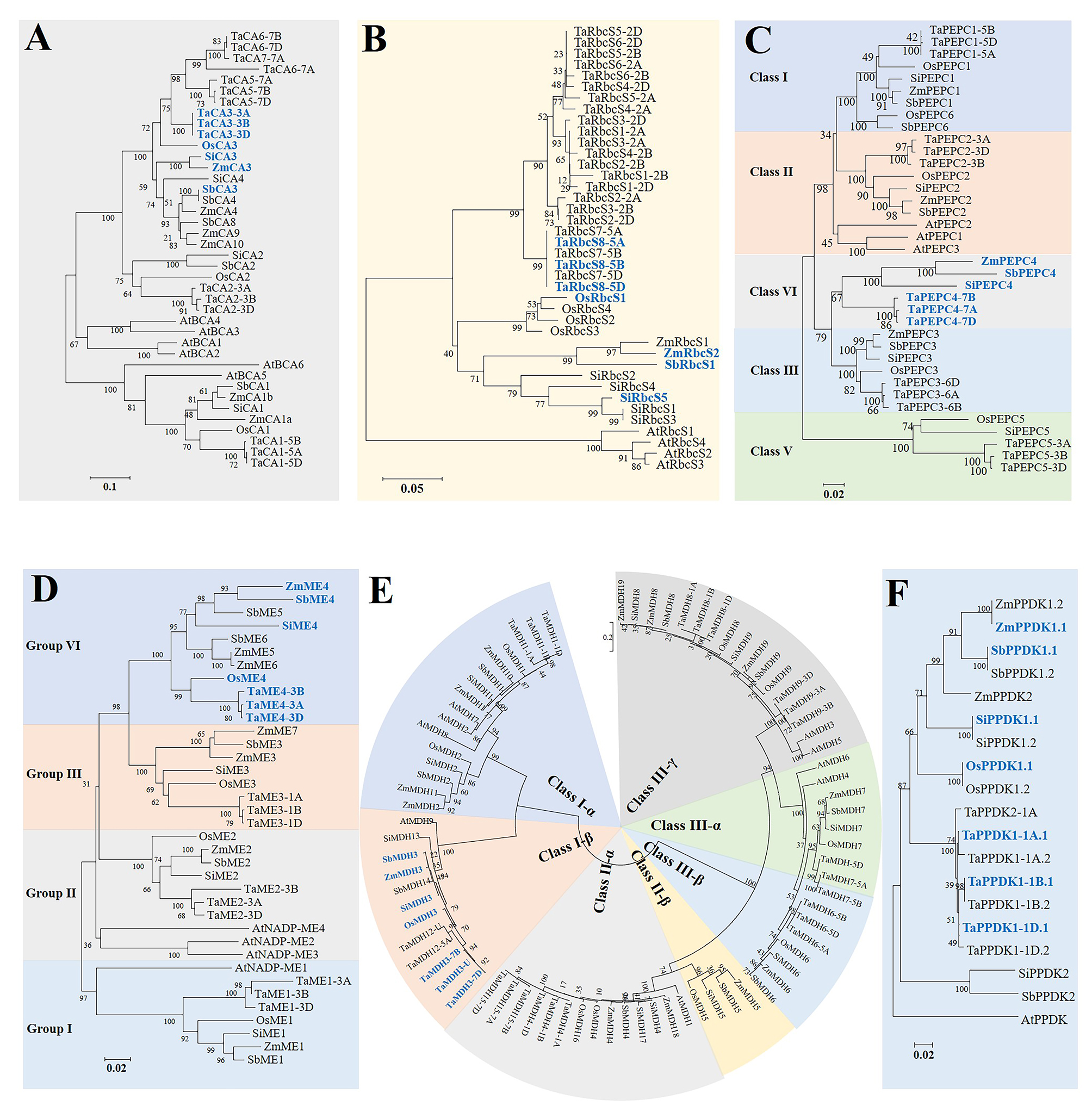

Supplement: Supplementary file 3 [file Image_1.jpeg]

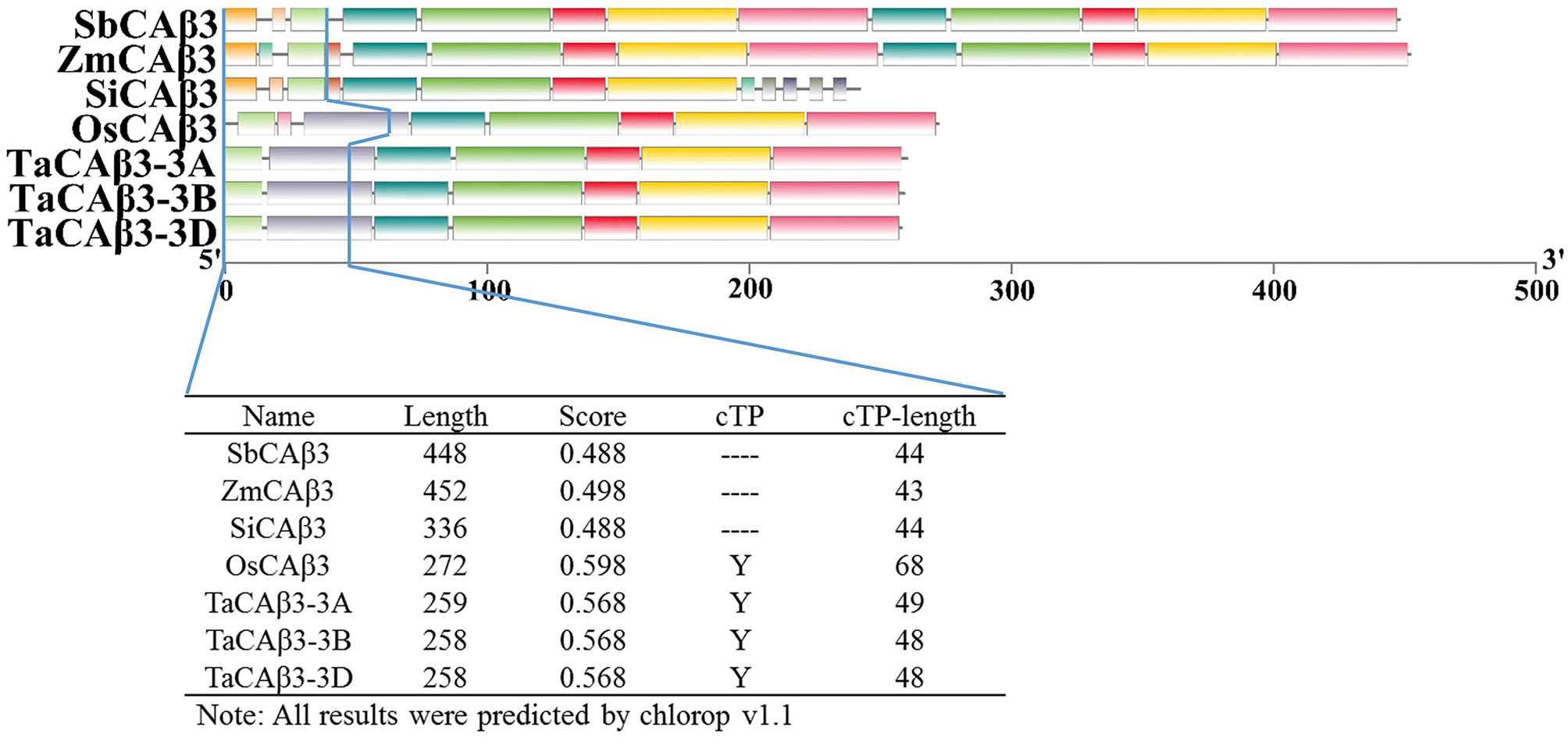

Supplement: Supplementary file 4 [file Image_2.jpeg]

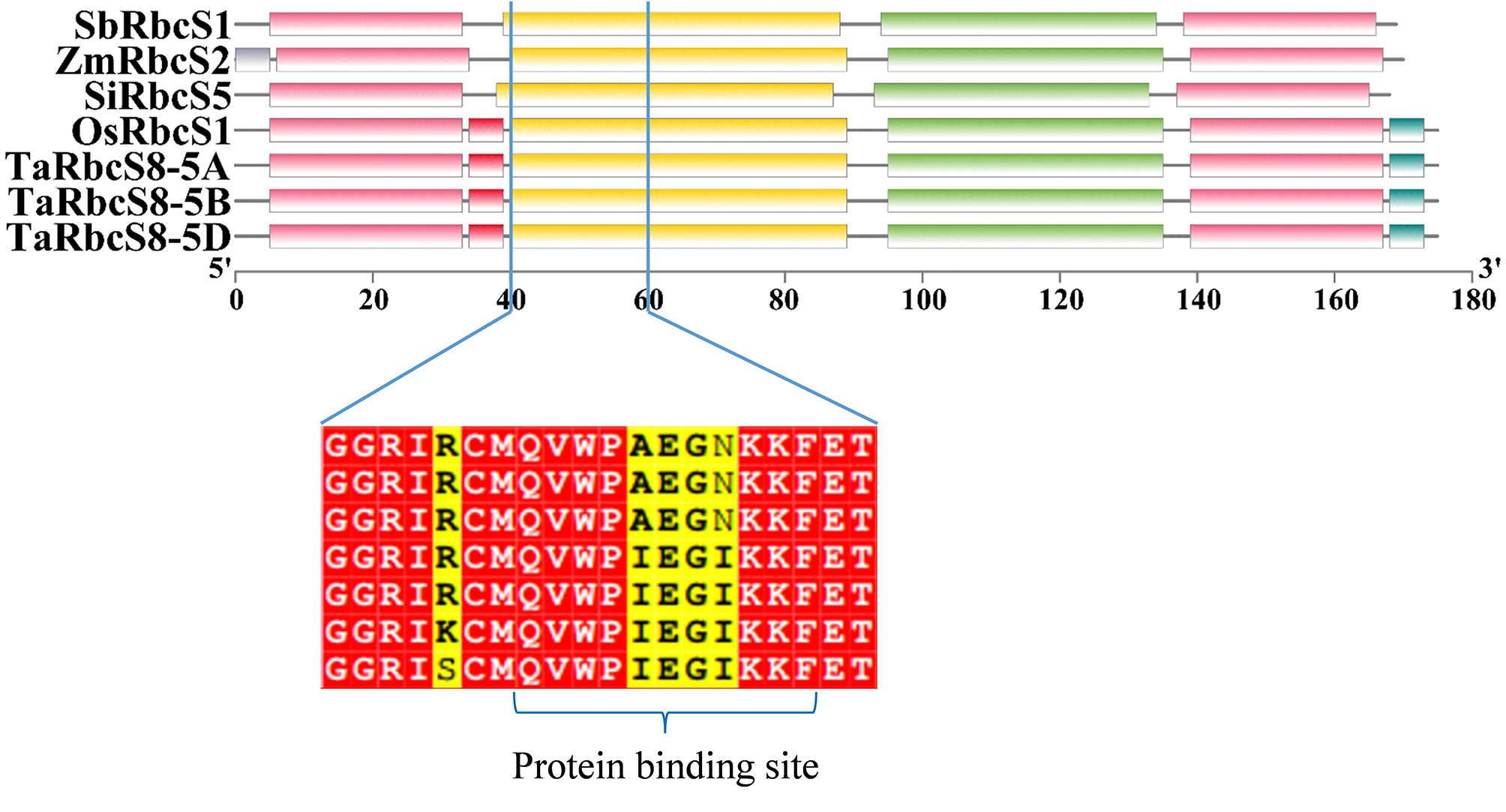

Supplement: Supplementary file 5 [file Image_3.jpeg]

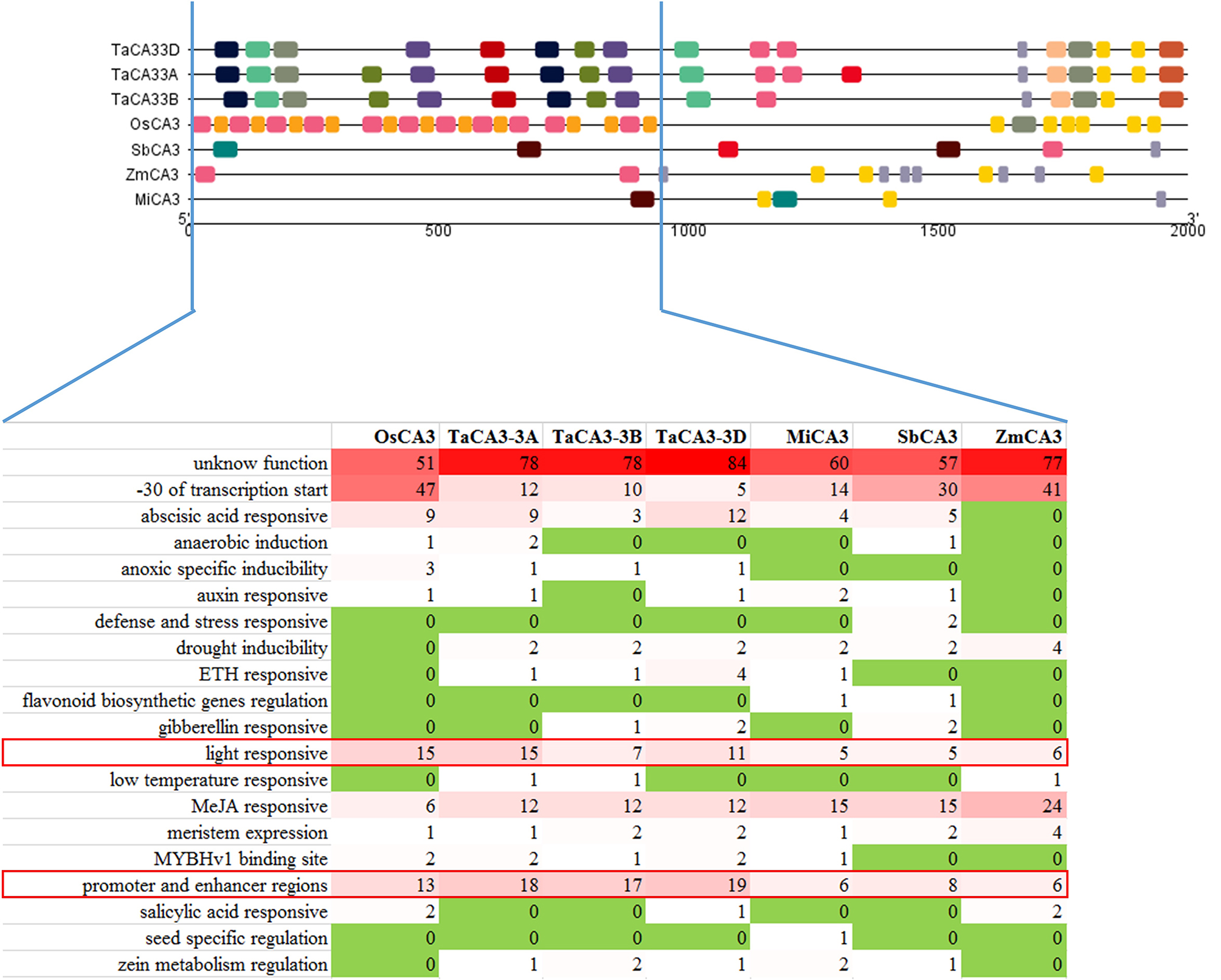

Supplement: Supplementary file 6 [file Image_4.jpeg]

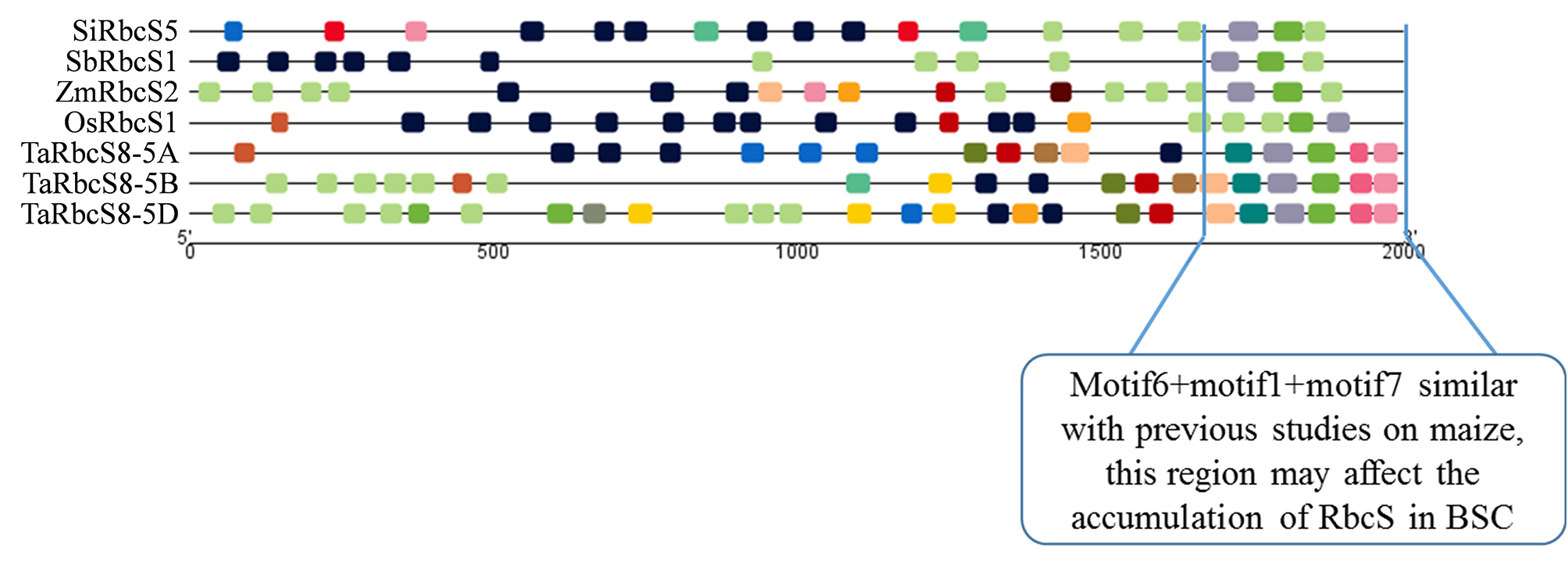

Supplement: Supplementary file 7 [file Image_5.jpeg]

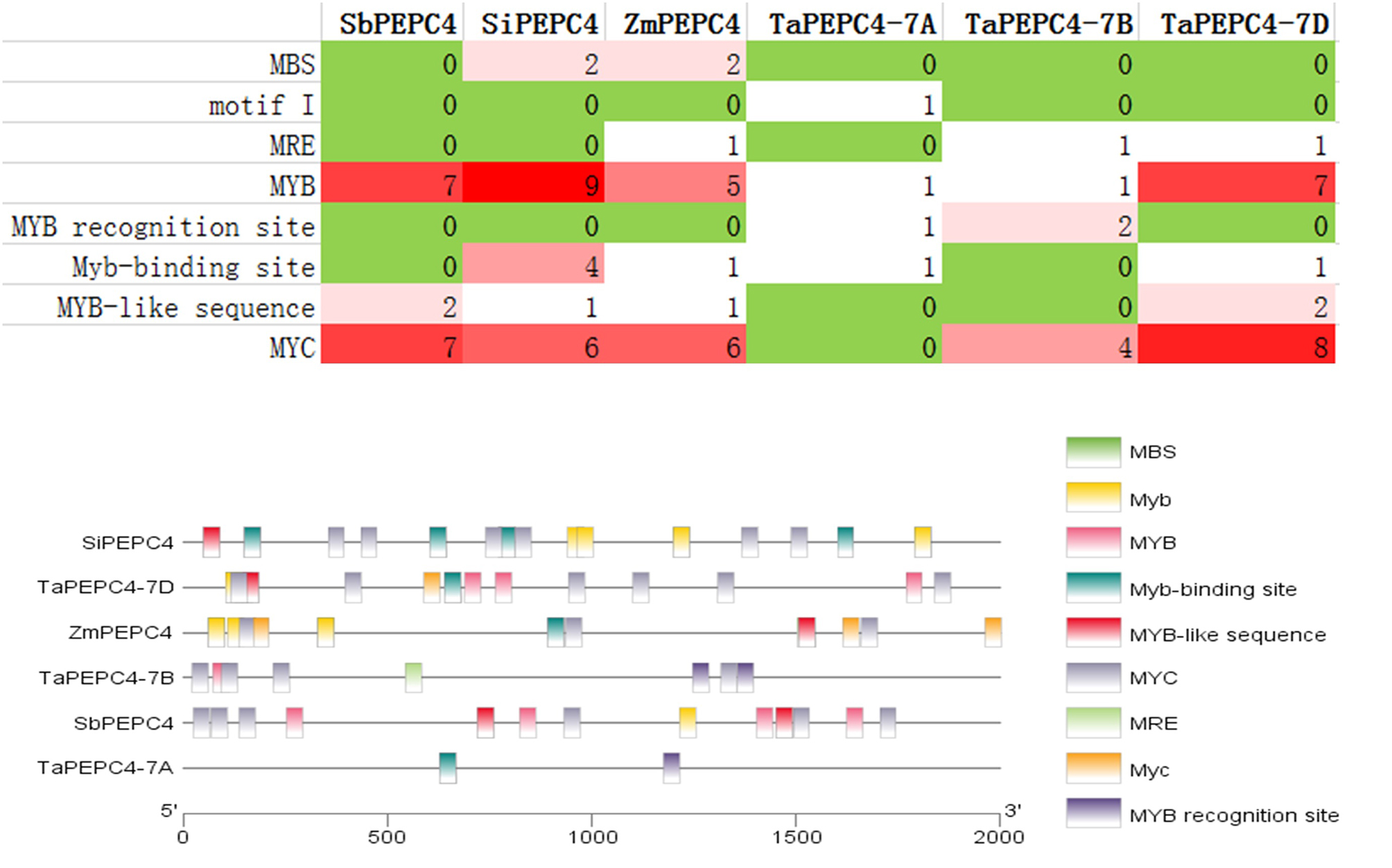

Supplement: Supplementary file 8 [file Image_6.jpeg]
